# Chokeberry (*A. melanocarpa* (Michx.) Elliott)—A Natural Product for Metabolic Disorders?

**DOI:** 10.3390/nu14132688

**Published:** 2022-06-28

**Authors:** Ewa Olechno, Anna Puścion-Jakubik, Małgorzata Elżbieta Zujko

**Affiliations:** 1 Department of Food Biotechnology, Faculty of Health Science, Medical University of Białystok, Szpitalna 37 Street, 15-295 Białystok, Poland; ewa.olechno@sd.umb.edu.pl (E.O.); malgorzata.zujko@umb.edu.pl (M.E.Z.); 2 Department of Bromatology, Faculty of Pharmacy with the Division of Laboratory Medicine, Medical University of Białystok, Mickiewicza 2D Street, 15-222 Białystok, Poland

**Keywords:** metabolic disorders, obesity, metabolic syndrome, diabetes, hyperglycemia, hypertension, dyslipidemia, inflammation

## Abstract

Abnormal metabolism of substances in the body can result in metabolic disorders which include obesity, cardiovascular diseases, diabetes, hypertension, chronic kidney disease, liver disease, or cancer. Foods rich in antioxidants can help to prevent and treat various types of disorders. Chokeberry fruits are rich in polyphenols, especially cyanidins, and therefore, can show a beneficial health effect. The aim of this study was to summarize and systematize reports about the effects of chokeberry on various metabolic parameters. Studies from 2000 to 2021, published in the PubMed and Google Scholar databases, were reviewed. The review of studies shows that chokeberry may have a positive effect in dyslipidemia and hypertension and may increase the body’s antioxidant defense mechanisms. The anti-inflammatory effect, in turn, may translate into a reduction in the risk of metabolic disorders over a longer period of use. Changes in glucose levels were reported by studies in which the intervention lasted more than 10 weeks in patients with carbohydrate metabolism disorders. The effects of protecting the liver, inhibiting platelet aggregation, lowering uric acid levels, and having a protective effect on the kidneys require additional confirmation in human clinical trials. Consumption of chokeberry fruit did not impact on anthropometric measurements; however, it seems that chokeberry fruit can be recommended in many metabolic disorders due to the richness of bioactive ingredients.

## 1. Introduction

Negative changes in the metabolism of substances in the body such as carbohydrates and lipids or inflammatory factors can result in metabolic disorders [1] which include obesity, insulin resistance, cardiovascular diseases, chronic kidney diseases, gastrointestinal diseases, liver diseases, neurodegenerative diseases, and cancer [2]. It seems that many of these disorders are influenced by the so-called Western lifestyle, including a diet high in calories and rich in processed foods, as well as low physical activity. Many of these disorders are interconnected and influence each other [3]. Obesity, that is, excess adipose tissue, plays a special role, which consequently contributes to the emergence of further disorders or disease entities [4]. According to the data of the World Health Organization from 2016, approximately 13% of the adult population (18 years or older) is obese (body mass index (BMI) > 30 kg/m^2^), while 39% of adults are overweight (BMI of 25–29.9 kg/m^2^) [5]. Visceral fat and, to a lesser extent, subcutaneous fat are metabolically active tissues, which in excess will interfere with the natural functioning of the body by secreting certain substances. Many of them are important for normal metabolism and food intake regulation. In some disorders, certain cell secretion signals are impaired, in other disorders, cells are resistant to the effects of some of these signals. Some of these are hormones, others are proinflammatory signals, among them are adiponectin, interleukin 6, tumor necrosis factor-α (TNFα), resistin, leptin, and angiotensinogen orplasminogen activator inhibitor-1 (PAI-1) [6]. Moreover, obesity and other metabolic disorders are often associated with chronic inflammation [1,7]. In 1988, Reaven described the phenomenon of the coexistence of various cardiovascular risk factors and proposed the definition of syndrome X [8]. Subsequently, this definition has been refined over the years as the metabolic syndrome (MetS) [9]. The MetS, also called insulin resistance syndrome, is a pathological condition that is a set of various risk factors for cardiovascular disease and type 2 diabetes [10]. The most recent definition of MetS has been proposed by the International Diabetes Federation (IDF) and the American Heart Association/National Heart, Lung and Blood Institute [11,12]. Actually, MetS is diagnosed when at least three of the following five risk factors have been identified: elevated waist circumference, elevated triglycerides, reduced HDL cholesterol, elevated blood pressure, and elevated fasting glucose [13]. 

MetS is diagnosed in one-third of the United States population and one-quarter of the European population [14,15]. Our previous study showed that MetS criteria were met by 36% of the Polish population [16].

Today, solutions are sought that could support the prevention and treatment of metabolic disorders. Particular attention is being given to products that are sources of antioxidants [17,18], for example, chokeberry fruit which can have a beneficial effect on health due to its high content of bioactive components, especially polyphenols [19,20,21]. In animal studies, chokeberry fruitt has been shown to have beneficial effects on certain metabolic disorders, such as antidiabetic [22,23,24], hepatoprotective [25,26,27,28,29], hypotensive [30,31], hypolipidemic [26,32,33,34,35], anti-inflammatory [36,37,38,39,40,41], antidepressant [42], anti-neurodegenerative [43,44,45], antibacterial [46], and antiviral [47] effects. Positive effects were also proven in two meta-analysis studies. The authors of these meta-analyses reported effects on lipid profile and blood pressure, but they did not observe an impact on some parameters of inflammation status [48,49].

Chokeberries, as compared with other berries, are distinguished by their particularly high antioxidant activity [50]. They contain flavonoids, including anthocyanins (constituting about 40% of total polyphenols), procyanidins (about 35% of total polyphenols), flavonols, and flavanols, as well as phenolic acids [51,52,53]. Among these, anthocyanins in chokeberry fruit provide a source of cyanidin-3-galactoside, cyanidin-3-glucoside, cyanidin-3-arabinoside, and cyanidin-3-xyloside [54]. Chokeberry phenolic acids include chlorogenic and neochlorogenic acids; flavanols, in particular epikatechins; and flavonols, in particular quercetin glycosides [54,55,56]. Among other substances, chokeberry fruit also contains vitamins (including vitamin C, B vitamins, carotenoids, vitamin K, and tocopherols), macroelements (calcium, magnesium, phosphorus, and potassium), and trace elements (copper, iodine, iron, manganese, zinc, and selenium) [51,57,58]. Chokeberry fruit is also a source of dietary fiber, including cellulose, hemicellulose, lignin, and pectins [51,52]. 

Fresh chokeberry fruit is characterized by a sour taste, and therefore, it are rarely eaten raw. Products made of chokeberry fruit include juices, dried fruit, fiber, jams, preserves, syrups, teas, tinctures, and powdered fruit in the form of supplements [19,51]. These products differ in the content of bioactive ingredients [19,56,59,60]. It is worth noting that the content of bioactive ingredients in chokeberry fruit may be influenced by various factors, including fruit variety, form of serving, harvest time, fruit ripeness, processing, and storage methods [19,21,53,54,56,61,62,63].

The aim of this review was to discuss the impact of consuming chokeberry fruit products in the form of juice or extract on selected metabolic disorders and to discuss the mechanisms of actions. This research is a compendium containing a summary of the current knowledge about the impact of chokeberry fruit on metabolic disorders. In this review, for the first time, we summarize and systematize the reports by numerous studies on the various effects of chokeberry fruit.

## 2. Materials and Methods

**Figure 1:**
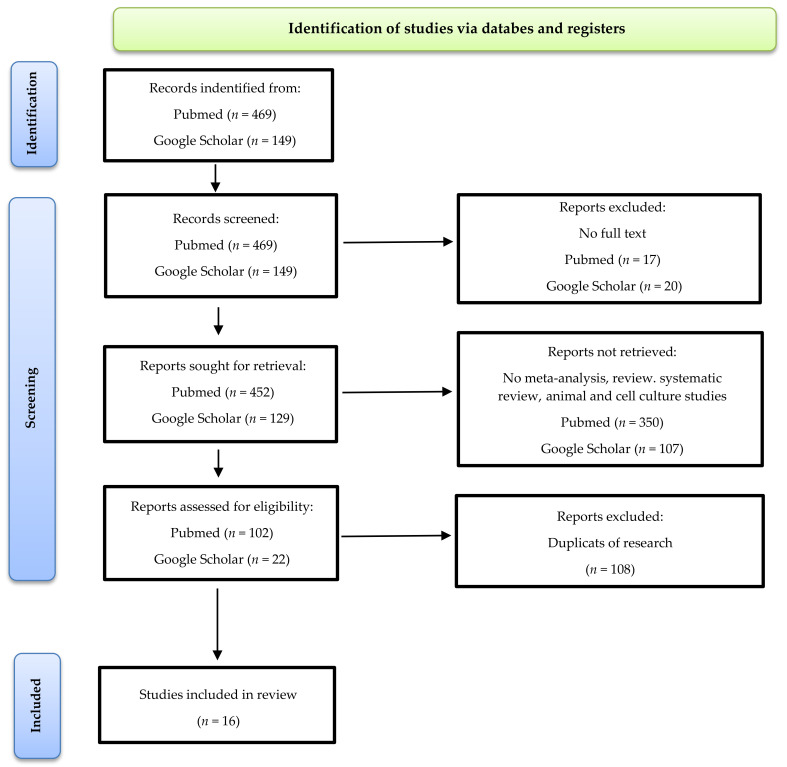
Literature review results, including exclusion factors.

In this study, we consider studies from 2000 to 2021. This time period was selected in order to narrow down the scope of the searched articles. The databases searched were Google Scholar and PubMed. The following terms were entered: ‘aronia melanocarpa’, ‘chokeberry’, ‘metabolic syndrome’, ‘obesity’, ‘diabetes’, ‘glucose’, ‘dyslipidemia’, ‘hyperlipidemia’, ‘lipid profile’, ‘cholesterol’, ‘blood pressue’, ‘hypertension’, ‘insulin resistance’, ‘inflammation’, ‘antioxidant status’, ‘anthropometric measurements’, ‘BMI’, ‘body weight’, ‘uric acid’, ‘creatinine’, ‘fibrinogen’, ‘kidney diseases’, ‘liver diseases’, and ‘blood clotting’. We included publications which contained information about the characterization of chokeberry products and an existing metabolic disorder (at least one). We excluded animal and cell line studies and all studies were in English. The exclusion criteria are shown in Figure 1.

## 3. Results and Discussion

Each subsection describes cell culture and animal research, while human clinical studies are summarized in the tables. The tables concern the influence of chokeberry fruit (juice or extract) on selected metabolic disorders.

Table 1, Table 2, Table 3, Table 4, Table 5, Table 6, Table 7, Table 8 and Table 9 provide an overview of the studies on metabolic disorders and the effects of chokeberry fruit on: anthropometric measurements (Table 1), carbohydrate metabolism (Table 2), blood pressure (Table 3), lipid profile (Table 4), inflammation and antioxidant status (Table 5 and Table 6), blood clotting (Table 7), liver functions (Table 8), uric acid, and creatinine level (Table 9). All information presented in the tables is statistically significant.

**Figure 2:**
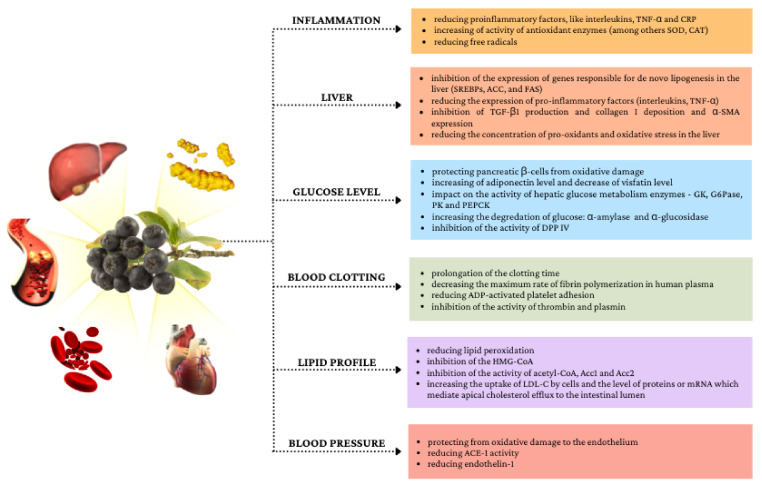
Selected mechanisms of the impact of chokeberry fruit on metabolic disorders.

Figure 2 shows selected mechanisms of the impact of chokeberry fruit on metabolic disorders.

Graphical markings in the tables in the form of arrows were used to better present the effects of chokeberry fruit on specific biochemical parameters.

### 3.1. Impact of Chokeberry on Anthropometric Measurements

Anthropometric measurements have been used to assess the nutritional status of children and adults. Among the anthropometric measurements, we can distinguish body mass index (BMI), circumference (waist, limbs, and hips), and the thickness of skin folds [64]. The body mass index takes into account both height and weight. A BMI score of of 25–29.9 is overweight, while a score of 30 and above is obesity [5]. It is well known that BMI scores are not always reliable markers of obesity, since this indicator does not reflect body fat content. Thus, people who do strength training, due to their high muscle mass, will have high BMI scores [65]. In 2009, during the unification of the metabolic syndrome criteria, waist circumference was considered to be an optional criterion and specific for ethnic groups [13]. According to The International Diabetes Federation criteria of metabolic syndrome from 2006, waist circumferences have not differed among women and men in Europid, Middle East, and Mediterranean Sub-Saharan African populations and were ≥80 cm for females and ≥94 cm for males. However, this indicator was lower for men in Asian, Ethnic Central, and South American populations (≥90 cm) [11].

The relationship between consumption of chokeberry juice/extract and anthropometric parameters seems to be unclear. It has been suggested that some polyphenols such as epigallocatechin gallates, resveratrol, and curcumin may affect cellular metabolism by modulating signaling pathways, including 5’ AMP-activated protein kinase (AMPK); inhibit the proliferation of preadipocytes and adipocytes; stimulate lipolysis and β-oxidation of fatty acids; and inhibit the accumulation of triglycerides [66]. A meta-analysis from 2018 showed that among the flavonoid subclasses, flavanols lowered BMI in whole populations and in Asian subgroups under 50 years of age, as well as decreased waist circumference [67]. However, in the case of anthocyanins, there have also been reports of their beneficial effect on body weight. Prior et al. (2008) showed that dietary supplementation of obese C57BL/6 mice fed a high-fat diet (60% energy from fat) and anthocyanin-rich berry extract from blueberries (2.9 mg/g of purified anthocyanins) decreased body weight and body fat as compared with a control group [68]. In another study, also in obese mice fed a high-fat diet, extracts of chokeberry, white mulberry, and a combination of chokeberry and mulberry for 14 weeks inhibited weight gain. Moreover, an increase in the expression of proteins related to AMPK signaling was observed in the case of combining the supply of chokeberry and white mulberry [69]. In a study by Kowalska et al. (2017), among other things, the effect of chokeberry extract on differentiated 3T3-L1 adipose cells was observed. The use of the extract in the amount of 100 μg/mL significantly decreased adipogenesis and lipogenesis. Moreover, it decreased leptin expression and increased adiponectin [70]. This may indicate a beneficial effect of chokeberry fruit on weight reduction.

Data from studies in humans are summarized in Table 1. In a study by Tasic et al. (2021), the authors observed decreased BMI in a group of men with metabolic syndrome (with diabetes) as well as decreased waist circumference (WC) and body weight (BW) in each group after 4 weeks of supplementation with chokeberry extract [71]. In turn, in a study by Kardum, Petrović-Oggiano et al. (2014) there was a decrease in WC, and BMI, and a downward trend in the case of BW [72]. Other studies in people with metabolic disorders reported no effect on anthropometric parameters [73,74,75,76,77,78,79]. In studies with healthy people, also no effect was seen [80]. 

It seems that consumption of chokeberry fruit in a short period of time (from 4 to 8 weeks) has no effect on anthropometric measurements. The overall daily diet and the caloric deficit in weight loss are very important. However, in the long term, chokeberry fruit may change the energy metabolism of adipose tissue by changing gene expression, which, so far, has only been proven in animal studies.

**Table 1:** Impact of chokeberry fruit on anthropometric measurements in intervention studies.

Number of Participants (*n*) (Women/Men)	Characteristics of the Group	Type of Chokeberry Product	Dose of Chokeberry Product per Day	Time of Intervention (Weeks)	Changes in Diet	Results	References
*n* = 44 (11/33)	Myocardial infarctionand statin therapy for at least 6 months (mean age 66, BMI 26.5 kg/m^2^)	Chokeberry flavonoid extract (Aronox, Agropharm, Pieńków, Poland)	3 × 85 mg	6	No changes	BMI↔	[78]
*n* = 47 (32/15)	MetS (*n* = 25, age 42–65, BMI 31.05 kg/m^2^) Healthy (*n* = 22, BMI 24.15 kg/m^2^)	Chokeberry extract (Aronox, Agropharm, Pieńków, Poland)	3 × 100 mg	8	No changes	BMI↔, WC↔	[77]
*n* = 52 (31/21)	MetS (*n* = 38, age 42–65, BMI 31.1 kg/m^2^), healthy (*n* = 14, age 42–65, BMI 24.4 kg/m^2^)	Chokeberry extract (Agropharm, Pieńków, Poland)	3 × 100 mg	8	Low-fat diet	BMI↔, WC↔	[74]
*n* = 70 (42/28)	Group I: patients with MetS who received chokeberry extract supplements (*n* = 25, age 50–69, BMI 30.9 kg/m^2^) Group II: healthy—control group (*n* = 45, age 55–71, BMI 23 kg/m^2^) Group III: patients with MetS treated with ACE inhibitors—control group (*n* = 25, age 50–69, BMI 29.2 kg/m^2^)	Chokeberry extract (Aronox, Agropharm, Pieńków, Poland)	3 × 100 mg	8	No changes (inhibition product containing chokeberry)	BMI↔, WC↔	[75]
*n* = 20 (20/0)	Postmenopausal women with abdominal obesity (WC > 88 cm, age 45–65, BMI 36.1 kg/m^2^)	Chokeberry supplement (Nutrika d.o.o., Belgrade, Serbia), prepared from pure chokeberry juice enriched with 2 g of stable glucomannan fibers (Luralean, Shimizu, Japan)	100 mL	4	No changes	BMI↓, WC↓ BW↔	[72]
*n* = 38 (24/14)	Mildly elevated BP, SBP 130–159 mmHg, DBP 85–99 mmHg (mean age 55.8, BMI < 35 kg/m^2^)	Cold-pressed 100% chokeberry juice (Kiantama Ltd., Suomussalmi, Finland) or convection oven-dried chokeberry powder (Finnish Berry Powders Ltd., Ähtäri, Finland)	300 mL chokeberry juice or 3 g dried chokeberry powder	8	No changes	BMI↔, BW↔	[76]
*n* = 84 (52/32)	Subjects with cardiovascular risks (mean age 40.6, BMI 27.29 kg/m^2^)	Chokeberry juice with a high-dose of polyphenols and chokeberry juice with a low-dose of polyphenols (Nutrika Ltd., Belgrade, Serbia)	100 mL	4	Avoiding excessive quantities of other foods rich in polyphenols	Low-dose of polyphenols group: BMI↔ High-dose of polyphenols group: BMI↔	[73]
*n* = 144 (74/70)	MetS according to the AHA guidelines (age 50–60, BMI 30.1–34.4 kg/m^2^) I. *n* = 42, fMetS II. *n* = 42, mMetS III. *n* = 32, fMetS-DM IV. *n* = 28, mMetS-DM	Standarized chokeberry extract (Alixir 400 PROTECT, Pharmanova, Belgrade, Serbia)	30 mL of extract (prior or during dinner)	4	No changes	fMetS: BW↓, WC↓, BMI↔ mMetS: BW↓, WC↓, BMI↔ fMetS-DM: BW↓, WC↓, BMI↔ mMetS-DM: BW↓, WC↓, BMI↓	[71]

↑—increase, ↓—decrease, ↔—no changes, BMI—body mass index; BP—blood pressure; BW—body weight; DBP—diastolic blood pressure; DM2—type 2 diabetes mellitus; fMetS—female with metabolic syndrome; fMetS-DM—female with metabolic syndrome and confirmed type 2 diabetes mellitus; MetS—metabolic syndrome; mMetS—male with metabolic syndrome; mMetS-DM—male with metabolic syndrome and type 2 diabetes mellitus; nd—no data; SBP—systolic blood pressure; WC—waist circumference.

### 3.2. Impact of Chokeberry on Carbohydrate Metabolism

It seems that chokeberry fruits, due to the fact that they are a rich source of anthocyanins as well as other polyphenols, can improve the parameters of carbohydrate metabolism [55,57]. The mechanisms of action of polyphenols, including anthocyanins, have been partially explored in animal and cellular models. As antioxidants, polyphenols help to fight reactive oxygen species. They can, therefore, be of special importance because diabetes is accompanied by an increase in reactive oxygen species caused by, among others, self-oxidation of glucose molecules and the formation of advanced glycation products [81]. Their metabolites may also play other roles in human cells such as influencing signaling pathways and there are also several other mechanisms that should be considered. Polyphenols reduce the absorption of glucose in the intestine and slow down the digestion of carbohydrates as a result of interaction with α-amylase (in the mouth and intestines) and α-glucosidase (in the intestine), as well as with sodium-dependent glucose transporter (SLGT1) [82,83,84]. Additionally, these compounds stimulate insulin secretion in the pancreas, activate insulin receptors, and regulate the release of glucose from the liver [83,85,86]. Polyphenols may also affect gene expression and intracellular signaling pathways [87].

In a 2017 study, a positive effect of 12 week of supplementation with purified anthocyanins was observed on glycemia in adults with prediabetes or early untreated type 2 diabetes mellitus (DM2). There was a decrease in glycosylated hemoglobin, an increase in adipsin, and a decrease in visfatin, which are associated with obesity and DM2, influencing, among other things, insulin sensitivity [88].

Another research also found a beneficial effect of anthocyanin supplementation purified from the bilberry and blackcurrant (total anthocyanin content was 80 mg/capsule) for 24 weeks on fasting plasma glucose, homeostatic model assessment of insulin resistance (HOMA-IR), and adiponectin levels [89]. Adiponectin is an adipokine which is secreted mainly by white adipose tissue. It plays an important role in increasing tissue sensitivity to insulin by enhancing the AMPK and peroxisome proliferator-activated receptor α (PPAR-α) pathways in skeletal muscle and liver [90]. Thus, it increases the oxidation of free fatty acids. Moreover, it exhibits anti-atherosclerotic and anti-inflammatory properties [91].

Hypoglycemic effects of chokeberry fruit were found in studies on animals [22,23,24]. Additionally, chokeberry fruits may have a beneficial effect by protecting pancreatic β-cells from oxidative damage [92]. In a study on diabetic mice, chokeberry juice inhibited α-glucosidase in the upper part of the small intestine, which contributed to a decrease in glucose levels [93]. The inhibitory effect on α-glucosidase was associated with the presence of anthocyanins, especially cyanidin-3-arabinoside [94]. Additionally, in a study by Kozuka et al. (2015), substances contained in chokeberry juice were found to inhibit the activity of another enzyme, i.e., dipeptidyl peptidase IV (DPP IV) in vitro. DPP IV is an enzyme that inactivates incretins such as glucagon-dependent insulinotropic polypeptide (GIP) and glucagon-like peptide-1 (GLP-1). This, in turn, leads to a decrease in insulin secretion [95]. Inhibitors of this enzyme are used in the treatment of DM2 [96]. The greatest inhibitory effect was noted with cyanidin-3,5-diglucoside as compared with cyanidin and cyanidin-3-glucoside [93,97].

Worsztynowicz et al. (2014) studied the effect of chokeberry extract on the activity of α-amylase and pancreatic lipase in vitro. Three types of chokeberry extracts (aqueous, methanolic, and acetic) inhibited the enzymes. After subjecting the extracts to reverse-phase preparative chromatography and high-performance liquid chromatography–mass spectrometry to determine the inhibitors, it was found that both anthocyanins and phenolic acids exhibited inhibitory properties against α-amylase and pancreatic lipase. Chlorogenic acid turned out to be the strongest pancreatic α-amylase inhibitor, and cyanidin-3-glucoside—pancreatic lipase [98]. Bräunlich et al. (2013) also confirmed that berries and bark extracts from chokeberry exhibited α-glucosidase inhibitory properties, especially procyanidin-enriched subfractions [94]. In another study, chokeberry extract served to rats with DM2 significantly reduced blood glucose and insulin levels. This effect was due to the influence on the activity of hepatic glucose metabolism enzymes, i.e., glucokinase, glucose-6-phosphatase, pyruvate kinase, and phosphoenolpyruvate carboxykinase. Additionally, chokeberry extract contributed to the activation of the phosphoinositide 3-kinase (PI3K)/protein kinase B (Akt) signaling pathway through, among other things, the elevation of insulin receptor substrate 2 (IRS-2) and glucose transporter 2 (GLUT 2) [99]. However, many mechanisms still need to be confirmed in human clinical trials.

Table 2 summarizes the studies conducted in humans. In the study by Tasic et al. (2021), the effects of supplementation with a standardized chokeberry extract (30 mL daily) on fasting glucose levels in patients with metabolic syndrome (MetS) without DM2 and with MetS connected with DM2 were investigated. The intervention lasted 4 weeks, while the level of glucose and other components was measured after 2 and 4 weeks of intervention. A significant effect of the extract on lowering glucose levels was observed in men and women with DM2, which, however, was due to higher glucose levels at the beginning of the study. The decrease in glucose after 4 weeks was over 14% in men with DM2 and approximately 24% in women with DM2. Men with MetS but without diabetes had a surprising increase in glucose levels: from 5.68 mmol/L to 6.99 mmol/L after 2 weeks and to 5.93 mmol/L after 4 weeks of intervention. In turn, in women with MetS without diabetes, a decrease of 0.07 mmol/L after 2 weeks of supplementation, while after 4 weeks the glucose level increased and was similar to the value before supplementation [71].

On the one hand, in a study by Broncel et al. (2010), the effect of 2 months of supplementation with chokeberry extract on fasting glucose levels in people with MetS was not observed [77].

On the other hand, a positive effect was observed in a study by Milutinović et al. (2019), where 3 months of consuming 150 mL of chokeberry juice daily by DM2 patients contributed to a decrease in fasting glucose and glycosylated hemoglobin levels (HbA1c). Interestingly, after the next 3 months, the decrease in glucose level continued, while the level of HbA1c returned to the value from before the treatment [100]. This may indicate a change in the diet by the participants of the study and a persistent elevated level of glucose, because glycosylated hemoglobin is formed as a result of glycation of globin, that is, the attachment of a glucose molecule to it [101].

The hypoglycemic effect of chokeberry was also noted by Lancrajan et al. (2012). In a patient with hypercholesterolaemia, hypertension, and deregulated protein metabolisms, there was a decrease from 102.88 mg% to 83 mg% of glucose level with an intake of 30 mL of chokeberry extract for 40 days. However, due to the fact that this study was conducted with only one patient, this publication can only guide the research by pointing to a positive effect. In addition, there is no information about the medications used by the patients and possible dietary changes [102].

Positive effects have also been observed in patients with hypercholesterolemia in a study by Skoczyńska et al. (2007) [103] and in overweight people in a study of Gancheva et al. (2021) [104]. In the study by Skoczyńska et al. (2014), four measurements were made during the intervention, i.e., before the start of the study, after a 6-week consumption, after a 6-week break in juice consumption, and again after 6 weeks of juice consumption. There was a decrease in fasting glucose levels from 99.3 mg/dL before the start of the study to 91.6 mg/dL after the end of the intervention [103]. In a study by Gancheva et al. (2021), the researchers assessed the impact of consuming 150 mL of chokeberry juice per day on various parameters of overweight people, but they did not have any other metabolic disorders. Overweight was defined as BMI of 25–29.9 kg/m^2^ and waist-to-height ratio of ≥0.49 for females and 0.53 for males. The researchers reported a decrease in fasting blood glucose (from 5.95 to 5.74 mmol/L) and HbA1c levels (from 5.70 to 5.47%) after 3 months of intervention [104].

In other studies taking into account carbohydrate metabolism, no effects of chokeberry (juice or supplement) were observed in people with metabolic disorders [72,76,78,79]. Interestingly, in a study by Yamane et al. (2017), they also observed a significant decrease in glucose levels in healthy people. Subjects consumed 100 mL of chokeberry juice before a meal of 200 g of rice as compared with a placebo. They measured the level of glucose before the meal and 30 min after drinking 100 mL of chokeberry juice or placebo. Next, the glucose level was measured 30, 45, 60, 75, 90, 120, and 150 min after eating the meal. In addition, researchers found a reduction in the levels of DPP IV and α-glucosidase enzymes by consuming chokeberry juice (40 µL) in vitro [105]. In other studies on healthy people, the effect of chokeberry juice was not observed [80,106,107].

Adiponectin also plays an important role in carbohydrate disorders. In a study by Naruszewicz et al. (2007), an increase in adiponectin was observed after 6 weeks of ingestion of chokeberry extract by people with myocardial infarction [78].

Changes in glucose levels were have been reported in studies in which the intervention lasted 10 weeks [102] or 12 weeks [100,103,104]. Only in a study by Lancrajan et al. (2012), 30 mL of alcoholic chokeberry extract (10%) was used [102], while in the other studies the amount of juice was from 100 to 300 mL daily [72,76,79,100,104]. HbA1c levels also decreased in studies by Gancheva et al. (2021) and Milutinović et al. (2019), in which the juice was administered for 12 weeks [100,104]. Surprisingly, adiponectin levels increased in a study by Naruszewicz et al. (2007) after just 6 weeks [78]. Only in a study by Tasic et al. (2021), a standardized chokeberry extract (30 mL/day) was used [71] in contrast to the other studies involving the extract without standardization [72,77,78,102]. The standardized extract was composed of polyphenols (431 mg/30 mL), anthocyanins (120 mg/30 mL), potassium sorbate (35.1 mg/30 mL), and low content of ethanol (0.04% *v*/*v*) [71].

In a study by Tasic et al. (2020), changes were observed after 4 weeks in people with MetS and coexisting diabetes [71]. Importantly, a decrease in glucose levels was noted in two studies involving people suffering from DM2, i.e., in studies by Milutinović et al. (2019) and Tasic et al. (2021) [71,100]. In studies by Gancheva et al. (2021), Kardum, Milovanović et al. (2015), and Naruszewicz et al. (2007), the exclusion criteria were not clearly defined, and people with carbohydrate metabolism disorders were not excluded, especially in the studies by Gancheva et al. (2021) and Naruszewicz et al. (2007), where the average glucose level was over 100 mg/dL in the study group before the intervention [78,79,104].

In addition, the content of polyphenols in a portion of the product may be important, but it is difficult to compare these data, because not all authors considered the content of polyphenols in a portion and used a different form of presenting the content of polyphenols. For example, in a study by Kardum, Petrović-Oggiano et al. (2014), the polyphenol content expressed in mg of gallic acid (GAE) was 586.7 mg/100 mL and in a study by Kardum, Milovanović et al. study (2015) it was 386 mg GAE/100 g juice [72,79]. This could have had an impact, among other factors, on the final result. It follows that both the longer use of 100% chokeberry juice (10 weeks or more) and the coexistence of type 2 diabetes mellitus may have a better effect of chokeberry on carbohydrate metabolism.

**Table 2:** Impact of chokeberry on carbohydrate metabolism in intervention studies.

Number of Participants (*n*) (Women/Men)	Characteristics of the Group	Type of Chokeberry Product	Dose of Chokeberry Product per Day	Time of Intervention (Weeks)	Changes in Diet	Results	References
*n* = 44 (11/33)	Myocardial infarction and statin therapy for at least 6 months (mean age 66, BMI 26.5 kg/m^2^)	Chokeberry flavonoid extract (Aronox, Agropharm, Pieńków, Poland)	3 × 85 mg	6	No changes	glucose ↔ adiponectin↑	[78]
*n* = 58 (0/58)	Mild hypocholesterolemia (TC > 200 mg/dL) without pharmacological treatment (mean age 54.1, BMI 27.7 kg/m^2^) without DM2	Organic chokebery juice (A. M. Lech, Dzieciolowo, Poland)	250 mL	18 (12 weeks with drinking chokeberry juice)	No changes	glucose↓	[103]
*n* = 47 (32/15)	MetS (*n* = 25, age 42–65, BMI 31.05 kg/m^2^) without DM2, healthy (*n* = 22, BMI 24.15 kg/m^2^)	Chokeberry extract (Aronox, Agropharm, Pieńków, Poland)	3 × 100 mg	8	No changes	glucose↔	[77]
*n* = 1	Hypercholesterolemia, arterial hypertension, and deregulated protein metabolism (67 years old) without DM2	Alcoholic extract of crude chokeberry fruits (10%)	30 mL	10	nd	glucose level↔	[102]
*n* = 20 (20/0)	Postmenopausal women with abdominal obesity (WC > 88 cm, age 45–65, BMI 36.1 kg/m^2^)	Chokeberry supplement (Nutrika d.o.o., Belgrade, Serbia), prepared from pure chokeberry juice enriched with 2 g of stable glucomannan fibers (Luralean, Shimizu, Japan)	100 mL	4	No changes	glucose↔	[72]
*n* =23 (11/12)	High normal BP or grade I hypertension: SBP = 130–159 mmHg, DBP = 85–99 mmHg, no regular use of antihypertensive drugs was declared by 23 patients (mean age 47.5, BMI nd)	Organic chokeberry juice (Conimex Trade d.o.o., Belgrade, Serbia)	200 mL	4	No changes	glucose↔	[79]
*n* = 38 (24/14)	Mildly elevated BP: SBP 130–159 mmHg, DBP 85–99 mmHg (mean age 55.8, BMI < 35 kg/m^2^) without DM2	Cold-pressed 100% chokeberry juice (Kiantama Ltd., Finland) or convection oven-dried chokeberry powder (Finnish Berry Powders Ltd., Finland)	300 mL chokeberry juice or 3 g dried chokeberry powder	8	No changes	glucose↔	[76]
*n* = 35 (23/12)	DM2 and oral antidiabetic drugs therapy for at least 6 months (mean age 56.3, BMI 28.8 kg/m^2^)	Chokeberry juice (Nutrica d.o.o., Belgrade, Serbia)	150 mL (three times daily for 50 mL)	12	No changes	glucose↓, HbA1c↓	[100]
*n* = 144 (74/70)	MetS according to the AHA guidelines (age 50–60, BMI 30.1–34.4 kg/m^2^) I. *n* = 42, fMetS II. *n* = 42, mMetS III. *n* = 32, fMetS-DM IV. *n* = 28, mMetS-DM	Standarized chokeberry extract (Alixir 400 PROTECT, Pharmanova, Belgrade, Serbia)	30 mL (prior or during dinner)	4	No changes	fMetS: glucose↔ mMetS: glucose↑ fMetS-DM: glucose↓ mMetS-DM: glucose↓	[71]
*n* = 22 (13/9)	Overweight (*n* = 11, mean age 51.9, BMI 25–30 kg/m^2^), healthy (*n* = 11, mean age 41.4, BMI 18–25 kg/m^2^)	Chokeberry juice (Aronia Alive Agriculture Ltd., Sofia, Bulgaria)	150 mL (50 mL, three times daily before meals)	12	nd	FPG↓, HbA1c↓	[104]

↑—increase, ↓—decrease, ↔—no changes, AHA—American Heart Association; BMI—body mass index; BP—blood pressure; DBP—diastolic blood pressure; DM2—type 2 diabetes mellitus; FBG—fasting plasma glucose; fMetS—female with metabolic syndrome; fMetS-DM—female with metabolic syndrome and confirmed type 2 diabetes mellitus; HbA1c—hemoglobin A1c; MetS—metabolic syndrome; mMetS—male with metabolic syndrome; mMetS-DM—male with metabolic syndrome and type 2 diabetes mellitus; nd—no data; SBP—systolic blood pressure; WC—waist circumference.

### 3.3. Impact of Chokeberry on Blood Pressure

According to the American Heart Association (AHA), hypertension can be diagnosed when a person’s systolic blood pressure (SBP) in the office or clinic is ≥140 mm Hg and/or their diastolic blood pressure (DBP) is ≥90 mm Hg, following repeated examination. The risk factors for hypertension include the presence in a patient’s history of: cardiovascular diseases, diabetes, dyslipidemia, kidney diseases, smoking, alcohol consumption, or inadequate diet, as well as genetic burden (family history of hypertension, hypercholesterolemia, diabetes, and CVD premature) [108]. Additionally, hypertension itself is also one of the most important risk factors for cardiovascular disease, stroke, and kidney failure [109]. The prevalence of hypertension is increasing worldwide. This is especially true of low and middle developing countries [110]. Reducing obesity and sodium intake and increasing the consumption of potassium or polyphenols in the diet may be important both in the prevention and in the treatment of hypertension [111,112].

According to a meta-analysis from 2019, the consumption of flavonoids was not associated with a reduction in the risk of hypertension, while in the case of the subclasses of these compounds, consumption of anthocyanins was associated with an 8% reduction in the risk of hypertension [113]. The beneficial effects of polyphenols are related to their antioxidant and anti-inflammatory effects, as well as their influence on regulatory pathways, for example, on the expression of specific genes [114]. The consumption of anthocyanins, apart from their antioxidant activity, may be associated with an increase in endothelial nitric oxide and a reduction in the production of vasoconstrictor molecules, for example, angiotensin II, endothelins, and thromboxanes [115]. In addition to providing polyphenols, chokeberry is also a source of essential potassium [116]. In people with metabolic syndrome, a decrease in systolic (SBP) and diastolic (DBP) pressure have been reported after intervention with chokeberry extract [71,75,77].

Table 3 summarizes the studies performed in people who consumed chokeberry juice or other products. In a study by Broncel et al. (2010), there were decreases in systolic blood pressure from 143.40 to 131.83 mmHg and in diastolic blood pressure from 87.2 to 82.13 mmHg, after 2 months of supplementation with chokeberry extract (100 mg three times daily) [77]. Sikora et al. (2014) observed decreases in SBP from 131.83 to 126.3 mmHg and DBP from 86.8 to 80.5 mmHg during interventions also with a chokeberry supplement (100 mg three times daily) [75]. Similar drops in SBP and DBP were noted by Tasic et al. (2021) [71]. In a study by Naruszewicz et al. (2007), chokeberry extract was also used in the amount of 255 mg per day for 6 weeks (3 × 85 mg/day). The extract with the declared composition was taken by people after myocardial infarction and being treated with statins. Statin therapy lasted at least 6 months (80% dose of 40 mg/day simvastatin). After 1.5 months, the SBP decreased by 11 mmHg (from 132.2 to 121.2 mmHg) and the DBP decreased by 7 mmHg (86.3 to 79.1 mmHg). The authors of the study emphasizes that chokeberry flavonoids may have dsignificantly reduced the risk of ischemic heart disease [78].

In patients diagnosed with hypertension, in a study by Kardum et al. (2015), chokeberry juice was used in the amount of 200 mL per day. After 4 weeks, there was a decrease in SBP during the 24-h measurement and in awake SBP and DBP, while sleep SBP and DBP remained unchanged [79]. In a study by Skoczyńska et al. (2007), SBP and DBP were decreased after 18 weeks of dietary intervention with chokeberry juice. The study included patients with mild hypercholesterolemia. Daily consumption of organic chokeberry juice in the amount of 250 mL lasted a total of 12 weeks. In the study, after 6 weeks of regular juice consumption, there was a 6-week juice break, and then chokeberry juice was reintroduced for another 6 weeks. Despite the interruption, the drop in blood pressure was sustained. Ultimately, the authors observed decreases in SBP from 138.6 to 125.1 mmHg and in DBP from 89 to 82 mmHg [103]. In turn, in a study by Loo et al. (2016), in patients with mild hypertension, there was only a statistically significant decrease in daytime DBP ambulatory after 8 weeks of consuming 300 mL organic chokeberry juice or 3 g dried chokeberry powder daily. In the remaining types of blood pressure (daytime SBP and 24-h SBP, awake, sleep and night DBP. and SBP), a downward but statistically insignificant trend (*p* > 0.05) was observed [76].

In another study by Kardum et al. (2014), 100 mL of pure chokeberry juice enriched with 2 g of stable glucomannan fibers was administered to postmenopausal women with abdominal obesity. The researchers only observed a decrease in SBP, while no change in DBP [72].

In patients with DM2 in a study by Milutinović et al. (2019), consumption of chokeberry juice in the amount of 150 mL/day did not bring any effects [100]. Pokimica et al. (2019) only observed the effect of 100 mL/day chokeberry juice with high-dose polyphenols after 4 weeks on SBP and DBP in people with cardiovascular risk factors [73]. Therefore, some polyphenols from chokeberry could play an important roles, but it is not known which polyphenolic compound exhibits the strongest action. A study by Tjelle et al. (2015) also confirmed hypotensive properties. This study was not included in this review due to the use of a mixture of juices from different fruits. Tjelle et al. (2015) studied the impact of: (1) commercial juice based on red grapes (67.7%), chokeberry (14.5%), cherry (12%) and bulberry (5.8%); (2) juice based on (1), but containing more polyphenols by adding polyphenol-rich extract from blackcurrant press residue (15%); and (3) a placebo. The study lasted 12 weeks and included 72 people (50–70 years) with pre-hypertension (130/85–139/89 mmHg) and 62 people with stage 1 and 2 hypertension (140/90–179/109 mmHg). The blood pressure was significantly reduced in the groups taking juice rich in polyphenols in people with hypertension. In people with pre-hypertension, the effect of juice was much weaker [117].

In studies with healthy people, chokeberry juice did not contribute to a statistically significant reduction in blood pressure [80,107] and in one study with the extract, only a reduction in DBP was observed [118]. Chokeberry juice or extract will probably not have a significant effect on the blood pressure of healthy people.

In hypertension, it is also worth mentioning the role of the angiotensin converting enzyme (ACE). It is responsible for regulating blood pressure and maintaining fluid homeostasis by influencing the renin-angiotensin-aldosterone and kallikrein-kinin systems. It is involved in the conversion of angiotensin I to angiotensin II, contributing to vasoconstriction and the release of aldosterone, and the breakdown of bradykinin. Thus, it causes an increase in blood pressure [119]. The evaluation of the activity of this enzyme can serve as an evaluation of the effectiveness of hypertension therapy with ACE inhibitors [120]. In a study by Sikora et al. (2014), supplementation with chokeberry extract contributed to a 25% decrease in ACE-1 activity after a month and a 30% decrease after 2 months of intervention in people with MetS who did not use antihypertensive and hyperlipidemic therapy [75]. In a study by Yamane et al. (2017), they also showed a decrease in ACE activity in vitro. Inhibition of activity was demonstrated at 95% by 40 µL of chokeberry juice [105]. When talking about blood pressure, it is worth mentioning endothelin-1 (ET-1). It is a substance which regulates various physiological processes such as vascular tone, and ET-1 neurotransmission is associated with the pathophysiology of heart disease [121,122]. Higher levels of ET-1 in plasma have been reported in people with congestive heart failure. This substance has a pro-arrhythmic effect, influencing the secretion of neurohormones such as vasopressin, adrenaline, and angiotensin II. It can also directly cause hypertrophy and fibrosis of the heart muscle [122]. In a study by Broncel et al. (2010), a decrease in ET-1 was reported in patients with MetS after 2 months of serving chokeberry extract in the form of a supplement [77].

Currently, it is known that chokeberry may have a positive effect on lowering blood pressure. Most studies have shown positive effects of consuming juice in the amount of 100–300 mL per day, as well as supplements in standardized and non-standardized forms. The exception was a study by Milutinović et al. (2019), in which no positive effect was observed [75]. The review showed that the positive effects of the juice on blood pressure could be observed after 4–12 weeks, and in the case of the extract, after 4–6 weeks.

**Table 3:** Impact of chokeberry on blood pressure in intervention studies.

Number of Participants (*n*) (Women/Men)	Characteristics of the Group	Type of Chokeberry Product	Dose of Chokeberry Product per Day	Time of Intervention (Weeks)	Changes in Diet	Results	References
*n* = 44 (11/33)	Myocardialinfarction and statin therapy for at least 6 months (mean age 66, BMI 26.5 kg/m^2^)	Chokeberry flavonoid extract (Aronox, Agropharm, Pieńków, Poland)	3 × 85 mg	6	No changes	SBP↓, DBP↓	[78]
*n* = 58 (0/58)	Mild hypocholesterolemia (TC > 200 mg/dL) without pharmacological treatment (mean age 54.1, BMI 27.7 kg/m^2^)	Organic chokebery juice (A. M. Lech, Dzieciolowo, Poland)	250 mL	18 (12 weeks with drinking chokeberry juice)	No changes	SBP↓, DBP↓	[103]
*n* = 47 (32/15)	MetS (*n* = 25, age 42–65, BMI 31.05 kg/m^2^), healthy (*n* = 22, BMI 24.15 kg/m^2^)	Chokeberry extract (Aronox, Agropharm, Pieńków, Poland)	3 × 100 mg	8	No changes	SBP↓, DBP↓ endthoteline-1↓	[77]
*n* = 70 (42/28)	Group I: patients with MetS who received chokeberry extract supplements (*n* = 25, age 50–69, BMI 30.9 kg/m^2^) Group II: healthy—control group (*n* = 45, age 55–71, BMI 23 kg/m^2^) Group III: patients with MetS treated with ACE inhibitors—control group (*n* = 25, age 50–69, BMI 29.2 kg/m^2^)	Chokeberry extract (Aronox, Agropharm, Pieńków, Poland)	3 × 100 mg	8	No changes (inhibition product containing chokeberry)	SBP↓, DBP↓, ACE↓	[75]
*n* = 20 (20/0)	Postmenopausal women withabdominal obesity (WC > 88 cm, age 45–65, BMI 36.1 kg/m^2^)	Chokeberry supplement (Nutrika d.o.o., Belgrade, Serbia), prepared from pure chokeberry juice enriched with 2 g of stable glucomannan fibers (Luralean, Shimizu, Japan)	100 mL	4	No changes	SBP↓, DBP↔	[72]
*n* = 23 (11/12)	High normal BP or grade I hypertension: SBP = 130–159 mmHg, DBP = 85–99 mmHg, no regular use of antihypertensive drugs (mean age 47.5, BMI nd)	Organic chokeberry juice (Conimex Trade d.o.o., Belgrade, Serbia)	200 mL	4	No changes	24 h SBP↓, 24 h DBP↔, awake SBP↓, awake DBP↓, sleep SBP↔, sleep DBP↔ 24 h pulse blood pressure↔, sleep pulse blood pressure↔, awake pulse blood pressure↓	[79]
*n* = 38 (24/14)	Mildly elevated BP: SBP 130–159 mmHg, DBP 85–99 mmHg (mean age 55.8, BMI < 35 kg/m^2^) without DM2	Cold-pressed 100% chokeberry juice (Kiantama Ltd., Finland) or convection oven-dried chokeberry powder (Finnish Berry Powders Ltd., Finland)	300 mL chokeberry juice or 3 g dried chokeberry powder	8	No changes	daytime ambulatory DBP↓ 24 h SBP/DBP↔, daytime ambulatory SBP↔, night SBP/DBP↔, awake SBP/DBP↔, sleep SBP/DBP↔	[76]
*n* = 35 (23/12)	DM2 and oral antidiabetic drugs therapy for at least 6 months (mean age 56.3, BMI 28.8 kg/m^2^)	Chokeberry juice (Nutrica d.o.o., Belgrade, Serbia)	150 mL (three times daily for 50 mL)	12	No changes	SBP↔, DBP↔	[100]
*n* = 84 (52/32)	Subjects with cardiovascular risks (mean age 40.6, BMI 27.29 kg/m^2^)	Chokeberry juice with a high dose of polyphenols and chokeberry juice with a low dose of polyphenols (Nutrika LTD, Belgrade, Serbia)	100 mL	4	Avoiding excessive quantities of other foods rich in polyphenols	low-dose of polyphenols group: SBP↔, DBP↔ high-dose of polyphenols group: SBP↓, DBP↓	[73]
*n* = 144 (74/70)	MetS according to the AHA guidelines (age 50–60, BMI 30.1–34.4 kg/m^2^) I. *n* = 42, fMetS II. *n* = 42, mMetS III. *n* = 32, fMetS-DM IV. *n* = 28, mMetS-DM	Standarized chokeberry extract (Alixir 400 PROTECT, Pharmanova, Belgrade, Serbia)	30 mL of extract (prior or during dinner)	4	No changes	fMetS: SBP↓, DBP↓, HR↔ mMetS: SBP↓, DBP↓, HR↔ fMetS-DM: SBP↓, DBP↓, HR↔ mMetS-DM: SBP↓, DBP↓, HR↔	[71]

↑—increase, ↓—decrease, ↔—no changes, ACE—angiotensin-converting enzyme; AHA—American Heart Association; BMI—body mass index; BP—blood pressure; DBP—diastolic blood pressure; DM2—diabetes mellitus 2; fMetS—female with metabolic syndrome; fMetS-DM—female with metabolic syndrome and confirmed type 2 diabetes mellitus; HR—heart rate; MetS—metabolic syndrome; mMetS—male with metabolic syndrome; mMetS-DM—male with metabolic syndrome and type 2 diabetes mellitus; nd—no data; SBP—systolic blood pressure; WC—waist circumference.

### 3.4. Impact of Chokeberry on Lipid Profile

Incorrect lipid profiles are mainly related to diets that are rich in saturated fatty acids, simple sugars, and processed foods, and also related to low physical activity [123,124]. Elevated levels of total cholesterol, low density lipoprotein cholesterol (LDL-cholesterol), and triglycerides are directly associated with increased cardiovascular risk and mortality [125]. Polyphenols such as flavonoids, lignans, and hydroxycinnamic/hydroxybenzoic acids can reduce lipase activity [126,127]; they can also modify lipid absorption in the intestine by influencing the lipid emulsification process, which has been shown with tea catechins [128]. Additionally, as demonstrated, flavanols, flavonols, flavones, isoflavones, flavanones, phenolic acids, anthocyanidins, and quercetin-3-*O*-rutinoside can bind to LDL at biologically relevant concentrations [129]. The action of anthocyanins, in turn, may be associated with lowering cholesterol levels by influencing the activity of AMPK [130]. AMPK inhibits the enzyme 3-hydroxy-3-methylglutaryl coenzyme A reductase (HMG-CoA), as well as the activity of acetyl-coenzyme A carboxylases ACC1 and ACC2. HMG-CoA is one of the main enzymes limiting cholesterol biosynthesis [131].

In addition, a reduction in the activity of carboxylases reduces the formation of fatty acids and, consequently, a decrease in triglycerides [132]. In addition, polyphenols have an antioxidant effect, and thus, reduce the risk of cholesterol oxidation [133,134]. Due to the high content of polyphenols, chokeberry fruit can modify the level of lipids. Its lipid-lowering effect has been confirmed in animal studies [24,32,33,34,35], which may be caused by, among other things, the influence on the expression of genes responsible for intestinal metabolism, which has been demonstrated in studies on Caco-2 cells of the intestine. Chokeberry extract decreases the expression of genes for 3-hydroxy-3-methylglutaryl coenzyme A reductase and sterol regulatory element binding protein 2 (responsible for cholesterol synthesis), Niemann-Pick C1-like1 and scavenger receptor class B type 1 (responsible for cholesterol uptake), and ATP-binding cassette transporter A1 (responsible for basolateral cholesterol efflux). Chokeberry extract has also been associated with increased uptake of LDL-cholesterol by cells and the level of proteins or mRNA which mediate apical cholesterol efflux to the intestinal lumen [135]. However, in one animal study, despite lowering cholesterol, no effect on the expression of genes responsible for cholesterol metabolism in the liver was reported [136].

Information on studies conducted in humans is provided in Table 4; During these studies, participants consumed various products from chokeberry. In studies involving patients with metabolic syndrome, changes in their lipid profiles were observed after supplementation with chokeberry extract, i.e., 100 mg of chokeberry extract, three times daily [74,75,77,137] or 30 mL of chokeberry extract once a day [71]. Results from these studies highlighted the following: a decrease in total cholesterol (TC) [71,74,75,77,137], LDL cholesterol (LDL-C) [71,74,75,77,137], and triglycerides (TG) [74,77,137] and an increase in plasma HDL cholesterol (HDL-C) [137]. Reductions in TC have been recorded at levels of about 5% to even approximately 27%. The largest decrease was observed by Tasic et al. (2021) in women with MetS and accompanying diabetes. This may also be due to the fact that the initial TC level was also high [71]. Duchnowicz et al. (2018), in turn, measured the level of cholesterol in erythrocytes and recorded a decrease of approximately 26% [137]. LDL-C in the abovementioned studies decreased from about 2.45 to 11% as compared with the initial value [71,74,75,77,137]. The greatest decrease in TG was reported in a study by Broncel et al. (2010), i.e., about 13% lower TG value after 2 months of supplementation with chokeberry extract [77]. HDL-C increased in a study by Duchnowicz et al. (2018) reported at around 3% as compared with baseline in people with MetS [137]. Positive changes in lipid profiles have been observed after 1 [71] or 2 months of supplementation [74,75,77,137].

In other studies, a positive effect of chokeberry on lipid metabolism was also observed in people with metabolic disorders. Milutinović et al. (2019) observed a statistically significant decrease in LDL-C, i.e., from 3.7 to 3.3 mmol/L and a decrease, but not statistically significant, in TC (from 6.1 to 5.7 mmol/L) and TG (from 2.2 to 1.9 mmol/L), as a result of 3 months of consuming chokeberry juice (150 mL/day), while after another 3 months without the juice, these values were similar to the initial state before the intervention. The HDL-C level did not change [100]. Duchnowicz et al. (2012) observed a decrease in TC from 4.85 to 3.58 mg/mL in patients with hypercholesterolemia after 2 months of intervention with chokeberry extract (300 mg daily) [137]. In a study by Lancrajan et al. (2012), a 40-day intervention with 30 mL of chokeberry extract contributed to a decrease in total cholesterol, LDL cholesterol, and triglycerides [102]. In a study by Skoczyńska et al. (2007), researchers also observed a decrease in TC, LDL-C, and TG in men with mild hypocholesterolemia [103]. In addition, there was a drop in plasma homocysteine levels from 9.4 to 8.8 µM/L.

On the other hand, in a study by Naruszewicz et al. (2007), there was no effect on the lipid profile or on the level of homocysteine. However, it is worth emphasizing that, in this study, statin therapy was used simultaneously, which could have distorted the results [78].

Homocysteine is an amino acid that plays a role in the metabolism of methionine. Its increased level is considered to be one of the risk factors for the development of cardiovascular diseases, due to redox imbalance [138]. According to a meta-analysis in 2018, elevated levels were associated with an increased risk of diabetic retinopathy, especially in patients with DM2 [139]. In a study from 2011, its level correlated with an increase in heart failure in patients after a myocardial infarction [140]. In another study from 2017, elevated homocysteine levels (≥12 µmol/L) were correlated with increased risk of long-term cardiovascular events in patients after coronary implantations of artery bare metal stents [141]. Lowering the level of homocysteine may be important in the prevention of cardiovascular diseases. In a study by Kardum, Milovanović et al. (2015), in patients with the presence of high normal BP or grade I hypertension, there was a decrease in TG from 1.95 to 1.57 mM, while there were no changes in TC, LDL-C, and HDL-C after 4 weeks of consuming 200 mL of organic chokeberry juice daily [79].

However, in a study by Kardum et al. (2014) on postmenopausal women with abdominal obesity, no significant changes in TC, TG, and LDL-C were reported after an intervention consisting of 100 mL daily of chokeberry juice enriched with 2 g of stable glucomannan fibers for 4 weeks. Surprisingly, a statistically significant decrease in HDL-C was observed, by about 6% as compared with the baseline value. The authors did not consider the possible cause of this phenomenon. In contrast, researchers have reported a statistically significant increase in n−3 polyunsaturated fatty acids in membrane phospholipids. Moreover, a significant increase in docosahexaenoic fatty acid, as well as a decrease in the n−6/n−3 ratio [72]. Increasing the level of omega-3 fatty acids may have a beneficial effect on reducing cardiovascular risk, as shown in a meta-analysis of 13 randomized, controlled trials with 127,477 participants in 2019 [142]. Similarly, in a study by Kardum et al. (2014), healthy individuals showed an increase in n−3 polyunsaturated fatty acids (PUFAs), total PUFAs, the C22/6n−3 ratio, and the n−6/n−3 ratio, as well as a decrease in monounsaturated fatty acids (MUFAs) and an increase in unsaturation index [80]. Pokimica et al. (2019) also found no effect on TC, LDL-C, HDL-C, and TG, but showed an increase in total n-6 polyunsaturated fatty acids, the n−6/n−3 ratio, and the arachidonic/eicosapenteonic acid ratio [73]. In other studies on overweight people and people with mildly elevated blood pressure, no changes in lipid profiles were found [76,104].

Loo et al. (2016) also studied the effect of juice consumption on the level of apolipoproteins A and B [76]. Apolipoproteins are proteins that are responsible for lipid binding. Apo-B is a building block of very low, low, and medium density lipoproteins and may reflect the amount of atherogenic molecules in the body. In turn, lipoprotein A is included mainly in high-density lipoproteins [143]. However, researchers have not reported the influence of chokeberry on these parameters.

In studies with healthy subjects, changes in plasma lipid levels have also been observed [106,144]. On the one hand, Petrovic et al. (2016) reported a decrease in TG in men (from 0.87 to 0.64 mmol/L), while in women an increase in TG (from 0.47 to 0.67 mmol/L), after 4 weeks. On the other hand, no changes in TC values were observed [106]. Xie et al. (2017) found a decrease in TC and LDL-C after 12 weeks of supplementation with chokeberry extract in healthy ex-smokers. Interestingly, the authors noted a correlation between the reduction in LDL-C and an increase in the urinary excretion of cyanidin-3-*O*-galactoside and its metabolites [144]. In the remaining studies on healthy subjects, no changes in cholesterol and triglyceride levels were observed after the intervention [80,107,145].

Apart from the values of lipid parameters, oxidized lipids also play a key role in the development of atherosclerosis [146]. Thiobarbituric acid reactive substances (TBARS) are a by-product of lipid peroxidation and are determined using thiobarbituric acid [147]. Duchnowicz et al. (2012) observed a decrease in lipid peroxidation from 0.464 to 0.281 µmol TBARS/g Hb, after 2 months of consuming chokeberry extract [148]. Similarly, in a study by Gancheva et al. (2021), TBARS value decreased after 3 months by 9.61 nmol/mL [104]. In turn, Naruszewicz et al. (2007) reported a decrease in oxidized cholesterol in people with myocardial infarction from 91.6 to 67.7 U/L, as measured by a non-competitive ELISA test [78].

A positive effect of chokeberry on a reduction in lipid peroxidation was also observed in healthy people [80,107,149]. In a study by Kardum et al. (2014), there was a decrease in plasma TBARS after 12 weeks of consuming chokeberry juice [80]. Similarly, Pilaczyńska-Szcześniak et al. (2005) observed a decrease in the TBARS index in rowers, both after training and in the 24-h recovery period [149]. In turn, in a study by Petrovic et al. (2016) on healthy male and female handball players, there was a statistically significant decrease in TBARS after 4 weeks in men, while there were no changes in women [106].

A study from 2021 assessed the effect of chokeberry juice consumption on the metalation of long interspersed nucleotide element 1 (LINE-1) in peripherial blood leukocytes in women with cardiovascular risk factors, including those who were overweight and dyslipidemic [150]. LINE-1 is considered to be an important factor in assessing the global methylation of the genome in the body. DNA methylation is an epigenetic process that reduces gene expression. LINE-1 methylation has been shown to be associated with metabolic disorders such as carbohydrate and lipid metabolism disorders [151,152,153]. Dietary components, including polyphenols, may influence DNA methylation [154,155]. Among the polyphenols influencing DNA methylation are catechin, epicatechin, (−)-epigallocatechin-3-*O*-gallate (EGCG), quercetin, and genistein [154,155]. Stojković et al. (2021) showed a decrease in LINE-1 methylation after 4 weeks of consuming chokeberry juice in women, and thus, as the authors emphasized, chokeberry may have had a cardioprotective effect [150].

Chokeberry fruit positively affects the levels of total cholesterol, LDL cholesterol, and triglycerides in the blood, as well as reduces levels of lipid peroxidation. The effects were reported both with the consumption of chokeberry juice (with the greatest hypolipidemic effect after a minimum of 12 weeks of consumption) and the consumption of chokeberry extract for a minimum period of 4 weeks. It is worth mentioning that the consumption of the juice after 4 weeks (150 mL daily) can lower the level of LDL-cholesterol oxidation. It seems that people with metabolic syndrome and hypercholesterolemia may benefit from consumption of chokeberry fruit.

**Table 4:** Impact of chokeberry on lipid profile in intervention studies.

Number of Participants (*n*) (Women/Men)	Characteristics of the Group	Type of Chokeberry Product	Dose of Chokeberry Product per Day	Time of Intervention (Weeks)	Changes in Diet	Results	References
*n* = 44 (11/33)	Myocardial infarction and statin therapy for at least 6 months in 44 patients (mean age 66, BMI 26.5 kg/m^2^)	Chokeberry flavonoid extract (Aronox, Agropharm, Pieńków, Poland)	3 × 85 mg	6	No changes	TC↔, LDL-C↔, HDL-C↔, TG↔, homocysteine↔	[78]
*n* = 58 (0/58)	Mild hypocholesterolemia (TC > 200 mg/dL) without pharmacological treatment (mean age 54.1, BMI 27.7 kg/m^2^)	Organic chokebery juice (A. M. Lech, Dzieciolowo, Poland)	250 mL	18 (12 weeks with drinking chokeberry juice)	No changes	TC↓ LDL-C↓ TG↓ homocysteine↓	[103]
*n* = 47 (32/15)	MetS (*n* = 25, age 42–65, BMI 31.05 kg/m^2^), healthy (*n* = 22, BMI 24.15 kg/m^2^)	Chokeberry extract (Agropharm, Pieńków, Poland)	3 × 100 mg	8	No changes	TC↓ LDL-C↓ TG↓	[77]
*n* = 45 (31/14)	Hypercholesterolemia without pharmacological treatment (*n* = 25, mean age 55.9,BMI nd), healthy (*n* = 20, mean age 50.3, BMI nd)	Chokeberry extract (Aronox, Agropharm, Pieńków, Poland)	3 × 100 mg	8	No changes	erythrocytes: TC↓ lipid peroxidation↓ TBARS↓	[148]
*n* = 1	Hypercholesterolemia, arterial hypertension, and deregulated protein metabolism (67 years old) without DM2	Alcoholic extract of crude chokeberry fruits (10%)	30 mL	10	nd	TC↓ LDL-C↓ HDL-C↔, TG↓	[102]
*n* = 52 (31/21)	MetS (*n* = 38, age 42–65, BMI 31.1 kg/m^2^), healthy (*n* = 14, age 42–65, BMI 24.4 kg/m^2^)	Chokeberry extract (Agropharm, Pieńków, Poland)	3 × 100 mg	8	Low-fat diet	TC↓, LDL-C↓, HDL-C↔, TG↓	[74]
*n* = 20 (2/0)	Postmenopausal women with abdominal obesity (WC > 88 cm, age 45–65, BMI 36.1 kg/m^2^)	Chokeberry supplement (Nutrika d.o.o., Belgrade, Serbia), prepared from pure chokeberry juice enriched with 2 g of stable glucomannan fibers (Luralean, Shimizu, Japan)	100 mL	4	No changes	TC↔, LDL-C↔, HDL-C↓, TG↔ membrane fatty acid profile in erythrocytes: SFA↔, MUFA, n-6 PUFA ↔, n-3 PUFA↑, total PUFA ↔, n-6/n-3↓ unsaturation index↑	[72]
*n* = 70 (42/28)	Group I: patients with MetS who received chokeberry extract supplements (*n* = 25, age 50–69, BMI 30.9 kg/m^2^) Group II: healthy—control group (*n* = 45, age 55–71, BMI 23 kg/m^2^) Group III: patients with MetS treated with ACE inhibitors—control group (*n* = 25, age 50–69, BMI 29.2 kg/m^2^)	Chokeberry extract (Aronox, Agropharm, Pieńków, Poland)	3 × 100 mg	8	No changes (inhibition product containing chokeberry)	TC↓, LDL-C↓, HDL-C↓	[75]
*n* = 23 (11/12)	High normal BP or grade I hypertension: SBP = 130–159 mmHg, DBP = 85–99 mmHg, no regular useof antihypertensive drugs (mean age 47.5 ± 10.4, BMI nd)	Organic chokeberry juice (Conimex Trade d.o.o., Belgrade, Serbia)	200 mL	4	No changes	TC↔ LDL-C↔ HDL-C↔ TG↓	[79]
*n* = 38 (24/14)	Mildly elevated BP: SBP 130–159 mmHg, DBP 85–99 mmHg (mean age 55.8, BMI <35 kg/m^2^) without DM2	Cold-pressed 100% chokeberry juice (KiantamaLtd, Finland) or convection oven-dried chokeberry powder (Finnish Berry Powders Ltd., Finland)	300 mL chokeberry juice or 3 g dried chokeberry powder	8	No changes	TC↔ HDL-C↔ TG↔ Apo-A1↔ Apo-B↔	[76]
*n* = 77 (40/37)	Children and adolescents (age 13–19) with MetS (modified criteria of the IDF)	Chokeberry extract (Aronox, Agropharm, Pieńków, Poland)	3 × 100 mg	8	No changes	TC↓ LDL-C↓ HDL-C↑ TG↓	[137]
*n* = 35 (23/12)	DM2 and oral antidiabetic drugs therapy for at least 6 months (mean age 56.3, BMI 28.8 kg/m^2^)	Chokeberry juice (Nutrica d.o.o., Belgrade, Serbia)	150 mL (three times daily for 50 mL)	12	No changes	TC↓ LDL-C↓ HDL-C↓	[100]
*n* = 84 (52/32)	Subjects with cardiovascular risks (mean age 40.6, BMI 27.29 kg/m^2^)	Chokeberry juice with a high dose of polyphenols and chokeberry juice with a low dose of polyphenols (Nutrika LTD, Belgrade, Serbia)	100 mL	4	Avoiding excessive quantities of other foods rich in polyphenols	low-dose of polyphenols group: TC↔, LDL-C↔, HDL-C↓, TG↔ high-dose of polyphenols group: TC↔, LDL-C↔, HDL-C↔, TG↔ oxLDL↓, oxLDL/TC ratio↓, oxLDL/LDL-C↓ changes in fatty acids in both groups: - PUFA n-6↑ - n6/n3 ratio (low-dose of polyphenols group)↑ - arachidonic/eicosapenteonic acid rate↑ - saturated fatty acids↑	[73]
*n* = 22 (13/9)	Overweight (n = 11, mean age 51.9, BMI 25–30 kg/m^2^), healthy (*n* = 11, mea n age 41.4, BMI 18–25 kg/m^2^)	Chokeberry juice (Aronia Alive Agriculture Ltd., Sofia, Bulgaria)	150 mL (50 mL—three times daily before meals)	12	nd	TC↔, LDL-C↔, HDLC↔, TG↔, TBARS↓	[104]
*n* = 144 patients (74/70)	MetS according to the AHA guidelines (age 50–60, BMI 30.1–34.4 kg/m^2^) I. *n* = 42, fMetS II. *n* = 42, mMetS III. *n* = 32, fMetS-DM IV. *n* = 28, mMetS-DM	Standarized chokeberry extract (Alixir 400 PROTECT, Pharmanova, Belgrade, Serbia)	30 mL (prior or during dinner)	4	No changes	fMetS: TC↓, LDL-C↓, HDL-C↑, TG↔ mMetS: TC↓, LDL-C↓, HDL-C↔, TG↔ fMetS-DM: TC↓, LDL-C↓, HDL-C↔,TG↓ mMetS-DM: TC↓, LDL-C↓, HDL-C↔, TG↓	[71]

↑—increase, ↓—decrease, ↔—no changes, ACE—angiotensin-converting enzyme; AHA—American Heart Association; Apo A-1—apolipoprotein A-1; Apo B—apolipoprotein B; BMI—body mass index; BP—blood pressure; DBP—diastolic blood pressure; DM2—type 2 diabetes mellitus; fMetS—female with metabolic syndrome; fMetS-DM—female with metabolic syndrome and confirmed type 2 diabetes mellitus; HDL—high-density lipoprotein; IDF—International Diabetes Federation; LDL—low-density lipoprotein; MetS—metabolic syndrome; mMetS—male with metabolic syndrome; mMetS-DM—male with metabolic syndrome and type 2 diabetes mellitus; nd—no data; oxLDL—oxidized low density lipoprotein; SBP—systolic blood pressure; TBARS—thiobarbituric acid reactive substances; TC—total cholesterol; TG—triglicerydes; WC—waist circumference.

### 3.5. Impact of Chokeberry on Inflammation and Antioxidant Status

Inflammation is a natural reaction of the immune system to various factors, usually harmful, including mechanical damage to tissues; viral, bacterial, or parasitic infections; chemical damage, for example, burns; as well as allergic or autoimmune reactions [156]. On the one hand, during acute inflammatory processes, harmful stimuli are removed and tissue repair processes are initiated. On the other hand, if the inflammatory process is prolonged, then a chronic inflammation occurs, and thus, a continuous generation of oxidative stress [157]. Chronic inflammation is significantly associated with many metabolic disorders [158] including atherosclerosis [159], ischemic heart disease [160], diabetes [161], liver diseases [162], kidney diseases [163], neurodegenerative diseases [164], and autoimmune diseases [165]. During this phenomenon, the activation of immunological processes takes place, including the main signaling pathways: nuclear factor kappa-light-chain-enhancer of activated B cells (NF-κB), mitogen-activated protein kinase (MAPK), and Janus kinase signal transducer and activator of transcription proteins (JAK-STAT) [166].

A balance in the body between pro-oxidative and antioxidant processes is extremely important. The body has natural enzymatic mechanisms, such as glutathione peroxidase or catalase, but with increased inflammatory processes it is important to supply antioxidants from the outside [167].

Chokeberry, due to its high content of polyphenols, may be one such product [51]. Inflammation in the body can be monitored by labeling specific biomarkers, such as acute phase proteins, mainly C-reactive protein (CRP); interleukins (IR); serum amyloid A; procalcitonin; cytokines, mainly tumor necrosis factor-α (TNFα); and interferon γ (IFNγ). Some of these biomarkers are disease specific [168]. Animal studies have shown that chokeberry can reduce inflammation as well as can improve antioxidant status [36,37,38,39,40,41]. In a study involving human cells, chokeberry extract inhibited the activation of the NF-κB pathway in RAW 264.7 macrophage cells and the release of interleukins IL-6 and IL-8 and TNFα in peripheral monocytes [169]. Another study on human endothelial cells showed possible anti-atherosclerotic effects of chokeberry. Chokeberry extract inhibited mRNA expressions of interleukins (IL-1β, IL-6, and IL-8) and monocyte chemoattractant protein-1 (MCP-1) upregulated by TNFα. Additionally, the extract inhibited the expression of vascular cell adhesion molecule-1 (VCAM-1), decreased monocyte/endothelial adhesion, as well as the signal transducer and activator of transcription proteins 3 (STAT-3)/interferon regulatory factor 1 (IRF-1) pathway. It contributed to a reduction in inflammation [170].

In a study by Loo et al. (2016), the effects of consuming 300 mL of 100% chokeberry juice/day or 3 g dried chokeberry powder/day on significant reductions in IL-10 (by 1.9 pg/mL) and TNFα (by 0.67 pg/mL) were reported in subjects with mildly elevated blood pressure. The authors of the study also emphasized that IL-4 and IL-5 showed a downward trend. However, the intervention did not reduce the remaining measured interleukins (IL-6, IL-7, IL-8, and IL-13) and granulocyte-macrophage colony-stimulating factor (GM-CSF) [76]. Naruszewicz et al. (2007) observed a decrease in high sensitivity C-reactive protein (hsCRP), high sensitivity interleukin-6 (hsIL-6), oxidized LDL (ox-LDL), soluble intercellular adhesion molecule-1 (S-ICAM), soluble vascular cell adhesion molecule-1 (S-VCAM), MCP-1, and F2-isoprostane, in people with myocardial infarction [78]. Elevated levels of adhesion molecules, i.e., S-ICAM and S-VCAM, indicate endothelial dysfunction and increase the risk of developing various pathologies, including atherosclerosis, cancer, or neurodegenerative diseases [171,172]. MCP-1 plays an important role in atherosclerosis, influencing the growth of other types of cells involved in the formation of atherosclerotic lesions [173]. Additionally, MCP-1 may bind to oxLDL and affect monocyte movement in atherosclerosis [174]. In turn, F2-isoprostane is formed during the peroxidation of arachidonic acid. They are important markers of oxidative damage [175,176]. At the same time, lowering these markers reduces the risk of metabolic disorders.

Tasic et al. (2021), in turn, determined the level of white blood cells (WBC), lymphocytes (LYM), and CRP after 2 and 4 weeks of taking a standardized chokeberry extract, in patients with metabolic syndrome without diabetes and with diabetes. After 2 weeks of supplementation, no statistically significant differences were observed in these parameters. The exception was a decrease in CRP in women with MetS, but without diabetes (from 5.8 to 2.8 mg/L), which did not change in the next measurement (after 2 weeks). In the remaining groups, a downward, but statistically insignificant, trend was observed after 2 weeks. In women and men without diabetes, a decrease in WBC levels was found (from 7.0 to 6.6 (109 cells/L) for women and from 8.9 to 7.0 (109 cells/L) for men) after 4 weeks. No changes in the LYM levels were observed in any of the studied groups [71]. In a study by Gancheva et al. (2021), the effect of consumption of juice for 3 months on a reduction in CRP of overweight people was observed (from 4.1 to 3.06 mg/L) [104]. In other studies involving MetS patients, no reducing effect of chokeberry extract on the level of CRP was found [75,77]. Changes in CRP were also not reported in patients with mild hypercholesterolemia [103], high normal or I degree hypertension [79], as well as in people with anemia and hemodialysis [177]. Taking into account healthy individuals, a study by Xie et al. (2017) reported that there was also no effect of chokeberry on the determined parameters of inflammation (CRP, IL-6, IL-1B, TNFα, adiponectin, vascular adhesion molecules, and chemokines) [144].

Maintaining the proper antioxidant status of the plasma and the activity of antioxidant enzymes is essential in reducing chronic inflammation. Food rich in antioxidants plays an important role [178]. Glutathione peroxidase (GPx) is an intracellular enzyme that reduces the level of hydrogen peroxide and reduces lipid peroxidation [179]. It is important in the development and prevention of chronic diseases [180]. A relationship has been shown between the consumption of chokeberry juice and an increase in the level of this enzyme in women with abdominal obesity [72] and in healthy subjects [107]. Pilaczyńska-Szcześniak et al. (2005) observed a decrease in GPx one minute after an activity test in healthy athletes after 4 weeks of daily consumption of chokeberry juice [149]. Other antioxidant enzymes that have been considered in studies involving chokeberry are superoxide dismutase (SOD) and catalase (CAT).

Milosavljevic et al. (2021) observed an increase in catalase and the level of reduced glutathione, which is a strong antioxidant [181], in hemodialysis patients after 30 days of consuming 30 mL of chokeberry extract daily [177]. In addition, they noted a decline in the plasma levels of the prooxidants: superoxide anion radical and nitrites. In a study by Gancheva et al. (2021), an increase in SOD activity was observed, while no change in CAT was observed, which was the opposite of the study by Milosavljevic et al. (2021), in which no changes in SOD were observed [104,177]. In studies with healthy people, both a decrease and no changes in SOD [72,107,144] and CAT were noted [72,144]. Pilaczyńska-Szcześniak et al. (2005) did not observe statistically significant changes in SOD, but 24 h after training, the enzyme activity after supplementation was slightly lower than before supplementation [149]. In a study by Nowak et al. (2016), a significant increase in blood antioxidant status was also observed in healthy people [145]. Kardum et al. (2014) did not observe changes in total oxidative status (TOS), while a decrease in total antioxidative capacity (TAC) and in pro-oxidant antioxidant balance (PAB) were reported [80].

Not all studies confirm the beneficial effect of chokeberry on inflammatory markers, mainly CRP. Similarly, in the case of antioxidant enzymes, the research is ambiguous. However, the effect of chokeberry seems to be promising due to its high antioxidant content, which is very important in the prevention and the treatment of metabolic disorders. This issue requires further research in humans.

**Table 5:** Impact of chokeberry on inflammation in intervention studies.

Number of Participants (*n*) (Women/Men)	Characteristics of the GROUP	Type of Chokeberry Product	Dose of Chokeberry Product per Day	Time of Intervention (Weeks)	Changes in Diet	Results	References
*n* = 44 (11/33)	Myocardial infarction and statin therapy for at least 6 months (mean age 66, BMI 26.5 kg/m^2^)	Chokeberry flavonoid extract (Aronox, Pieńków, Agropharm, Poland)	3 × 85 mg	6	No changes	hsCRP↓, hsIL-6↓, ox-LDL↓, S-ICAM↓, S-CAM↓, MCP-1↓, F1-isoprostane↓	[78]
*n* = 58 (0/58)	Mild hypocholesterolemia (TC > 200 mg/dL) without pharmacologicaltreatment (mean age 54.1, BMI 27.7 kg/m^2^)	Organic chokebery juice (A. M. Lech, Dzieciolowo, Poland)	250 mL	18 (12 weeks with drinking chokeberry juice)	No changes	CRP↔	[103]
*n* = 47 (32/15)	MetS (*n* = 25, age 42–65, BMI 31.05 kg/m^2^), healthy (*n* = 22, BMI 24.15 kg/m^2^)	Chokeberry extract (Aronox, Agropharm, Pieńków, Poland)	3 × 100 mg	8	No changes	CRP↔	[77]
*n* = 70 (42/28)	Group I: patients with MetS who received chokeberry extract supplements (*n* = 25, age 50–69, BMI 30.9 kg/m^2^) Group II: healthy—control group (*n* = 45, age 55–71, BMI 23 kg/m^2^) Group III: patients with MetS treated with ACE inhibitors—control group (*n* = 25, age 50–69, BMI 29.2 kg/m^2^)	Chokeberry extract (Aronox, Agropharm, Pieńków, Poland)	3 × 100 mg	8	No changes (inhibition product containing chokeberry)	CRP↔	[75]
*n* = 23 (11/12)	High normal BP or grade I hypertension: SBP = 130–159 mmHg, DBP = 85–99 mmHg, no regular use of antihypertensive drugs (mean age 47.5, BMI nd)	Organic chokeberry juice (Conimex Trade d.o.o., Belgrade, Serbia)	200 mL	4	No changes	CRP↔	[79]
*n* = 38 (24/14)	Mildly elevated BP: SBP 130–159 mmHg, DBP 85–99 mmHg (mean age 55.8, BMI < 35 kg/m^2^) without DM2	Cold-pressed 100% chokeberry juice (Kiantama Ltd, Finland) or convection oven-dried chokeberry powder (Finnish Berry Powders Ltd., Finland)	300 mL chokeberry juice or 3 g dried chokeberry powder	8	No changes	IL-10↓, TNFα↓, IL-4-↔, IL-5↔, IL-6↔, IL-7↔, IL-8↔, IL-13↔, GM-CSF↔	[76]
*n* = 35 (23/1)	DM2 2 and oral antidiabetic drugs therapy for at least 6 months (mean age 56.3, BMI 28.8 kg/m^2^)	Chokeberry juice (Nutrica d.o.o., Belgrade, Serbia)	150 mL (three times daily for 50 mL)	12	No changes	WBC↓, LYM↓, CRP↔	[100]
*n* = 22 (13/9)	Overweight (*n* = 11, mean age 51.9, BMI 25–30 kg/m^2^), healthy (*n* = 11, mean age 41.4, BMI 18–25 kg/m^2^)	Chokeberry juice (Aronia Alive Agriculture Ltd., Sofia, Bulgaria)	150 mL (50 mL, three times daily before meals)	12	nd	CRP↓	[104]
*n* = 30 (11/19)	Anemia: Hb < 110 g/L, and hemodialysis >3 months, >3 times week (mean age 62.93, BMI 25.82 kg/m^2^)	Polyphenol-rich standardized chokeberry extract (EU-Chem Company, Belgrade, Serbia)	30 mL	4	nd	CRP↔, leukocytes↔, TNFα↔	[177]
*n* = 144 (74/70)	MetS according to the AHA guidelines (age 50–60, BMI 30.1–34.4 kg/m^2^) I. *n* = 42, fMetS II. *n* = 42, mMetS III. *n* = 32, fMetS-DM IV. *n* = 28, mMetS-DM	Standarized chokeberry extract(Alixir 400 PROTECT, Pharmanova, Belgrade, Serbia)	30 mL (prior or during dinner)	4	No changes	fMetS: WBC↓, CRP↓, LYM↔ mMetS: WBC↓, CRP↔, LYM↔ fMetS-DM: WBC↔, CRP↔, LYM↔ mMetS-DM: WBC↔, CRP↔, LYM↔	[71]

↑—increase, ↓—decrease, ↔—no changes, ACE—angiotensin-converting enzyme; AHA—American Heart Association; BMI—body mass index; BP—blood pressure; CRP—C-reactive protein; DBP—diastolic blood pressure; DM2– type 2 diabetes mellitus; fMetS—female with metabolic syndrome; fMetS-DM—female with metabolic syndrome and confirmed type 2 diabetes mellitus; GM-CSF—granulocyte-macrophage colony-stimulating factor; Hb—hemoglobin; hsCRP—high sensitivity C-reactive protein; hsIL-6—high sensitivity interleukin 6; IL—interleukin; LYM—lymphocytes; MCP-1—monocyte chemoattractant protein-1; MetS—metabolic syndrome; mMetS—male with metabolic syndrome; mMetS-DM—male with metabolic syndrome and type 2 diabetes mellitus; nd—no data; oxLDL—oxidized low density lipoprotein; SBP—systolic blood pressure; S-ICAM—soluble intercellular adhesion molecule-1; S-VCAM—soluble vascular cell adhesion molecule-1, TNFα—tumor necrosis factor-α; WBC—white blood cells.

**Table 6:** Impact of chokeberry on antioxidant status in intervention studies.

Number of Participants (*n*) (Women/Men)	Characteristics of the Group	Type of Chokeberry Product	Dose of Chokeberry Product per Day	Time of Intervention (Weeks)	Changes in Diet	Results	References
*n* = 20 (20/0)	Postmenopausal women withabdominal obesity (WC > 88 cm, age 45–65, BMI 36.1 kg/m^2^)	Chokeberry supplement (Nutrika d.o.o., Belgrade, Serbia), prepared from pure chokeberry juice enriched with 2 g of stable glucomannan fibers (Luralean, Shimizu, Japan)	100 mL	4	No changes	GSH-Px↑, SOD↔, CAT↔	[72]
*n* = 22 (13/9)	Overweight (*n* = 11, mean age 51.9, BMI 25–30 kg/m^2^), healthy (*n* = 11, mean age 41.4, BMI 18–25 kg/m^2^)	Chokeberry juice (Aronia Alive Agriculture Ltd., Sofia, Bulgaria)	150 mL (50 mL—three times daily before meals)	12	nd	SOD↑ CAT↔	[104]
*n* = 30 (11/19)	Anemia: Hb < 110 g/L and hemodialysis >3 months, >3 times week (mean age 62.93, BMI 25.82 kg/m^2^)	Polyphenol-rich standardized chokeberry extract (EU-Chem Company, Belgrade, Serbia)	30 mL	4	nd	CAT↑, reduced glutathione↑superoxide anion↓, radical↓, nitrite↓ hydrogen peroxide↔, SOD↔	[177]

↑—increase, ↓—decrease, ↔—no changes, BMI—body mass index; CAT—catalase; GSH-Px—plasma glutathione peroxidase; Hb—hemoglobin; nd—no data; SOD—superoxide dismutase; WC—waist circumference.

### 3.6. Impact of Chokeberry on Blood Clotting

Blood coagulation is associated with a greater risk of atherosclerosis, which in turn may lead to heart disease [182]. A number of proteins and coagulation factors are involved in the coagulation process, and almost all of them are also present in atherosclerotic lesions [182,183]. It seems that flavonoids and isoflavones can act favorably and can reduce blood clotting [184]. In a study by Bijak et al. (2011) in vitro, chokeberry extract prolonged the clotting time and decreased the maximum rate of fibrin polymerization in human plasma [185]. In a study by Malinowska et al. (2012), the effect of chokeberry extract on clot formation using human plasma and purified fibrinogen as well as fibrin lysis in an in vitro hyperhomocysteinemia model was assessed. A positive effect of chokeberry extract on hemostatic properties of fibrinogen or plasma was observed [186]. Another in vitro study also found a beneficial effects of chokeberry extract on reduced adenosine diphosphate (ADP)-activated platelet adhesion and inhibition of the amidolytic activity of thrombin and plasmin [187].

Human studies have also shown promising results. Sikora et al. (2012) examined various parameters related to clotting processes, and the most important, in our opinion, are summarized in Table 7, i.e., platelet aggregation, fibrinolysis, and clot preparation. They observed inhibited platelet aggregation after a month of supplementation with chokeberry extract in people with MetS. Inhibition of platelet aggregation was reduced in maximal aggregation and initial velocity of the process but also an increase in the time after which maximal aggregation was reached. In addition, a decrease in overall clot formation and fibrinolysis was observed after one month. However, after 2 months, these effects became less visible as compared with the values before supplementation [74].

In a study by Broncel et al. (2010), monthly supplementation with chokeberry extract in patients with MetS had no effect on the level of fibrinogen, while a significant increase was observed after two months [77]. In a study by Skoczyńska et al. (2007) in people with mild hypocholesterolemia, a decrease in fibrinogen was observed as a result of consuming organic chokeberry juice [103]. In a study by Loo et al. (2016), no effect of consuming 300 mL juice/day or 3 g dried chokeberry powder/day on platelet aggregation as measured by collagen. and adenosine diphosphate closure time (CADP-CT) and collagen and epinephrine closure time (CEPI-CT) were observed [76]. Surprisingly, Tasic et al. (2021) observed an increase in platelets level (PLT) after 4 weeks of juice consumption in diabetes patients [71]. In studies on healthy runners, a positive effect of chokeberry juice was reported, which, thanks to the content of polyphenols, could counteract platelet aggregation caused by a half-marathon. The expressions of clotting proteins, P-selectin and GPIIb-IIIa expressions, were significantly reduced in the chokeberry juice group as compared with a placebo [188].

The effect of chokeberry on blood clotting is promising but requires further human research due to insufficient scientific evidence supporting this effect.

**Table 7:** Impact of chokeberry on blood clotting in intervention studies.

Number of Participants (*n*) (Women/Men)	Characteristics of the Group	Type of Chokeberry Product	Dose of Chokeberry Product per Day	Time of Intervention (Weeks)	Changes in Diet	Results	References
*n* = 58 (0/58)	Mild hypocholesterolemia (TC > 200 mg/dL) without pharmacological treatment (mean age 54.1, BMI 27.7 kg/m^2^)	Organic chokebery juice (A. M. Lech, Dzieciolowo, Poland)	250 mL	18 (12 weeks with drinking chokeberry juice)	No changes	fibrinogen↓	[103]
*n* = 47 (32/15)	MetS (*n* = 25, age 42–65, BMI 31.05 kg/m^2^), healthy (*n* = 22, BMI 24.15 kg/m^2^)	Chokeberry extract (Agropharm, Pieńków, Poland)	3 × 100 mg	8	No changes	fibrinogen↑	[77]
*n* = 52 (31/21)	MetS (*n* = 38, age 42–65, BMI 31.1 kg/m^2^), healthy (*n* = 14, age 42–65, BMI 24.4 kg/m^2^)	Chokeberry extract (Agropharm, Pieńków, Poland)	3 × 100 mg	8	Low-fat diet	platelet aggregation↔, fibrinolysis↔, clot preparation ↔	[74]
*n* = 38 (24/14)	Mildly elevated BP: SBP 130–159 mmHg, DBP 85–99 mmHg (mean age 55.8, BMI < 35 kg/m^2^) without DM2	Cold-pressed 100% chokeberry juice (KiantamaLtd, Finland) or convection oven-dried chokeberry powder (Finnish Berry Powders Ltd., Finland)	300 mL of chokeberry juice or 3 g of dried chokeberry powder	8	No changes	CEPI-CT↔, CADP-CT↔, PLT↔	[76]
*n* = 144 patients (74/70)	MetS according to the AHA guidelines (age 50–60, BMI 30.1–34.4 kg/m^2^) I. *n* = 42, fMetS II. *n* = 42, mMetS III. *n* = 32, fMetS-DM IV. *n* = 28, mMetS-DM	Standarized chokeberry extract (Alixir 400 PROTECT, Pharmanova, Belgrade, Serbia)	30 mL (prior or during dinner)	4	No changes	fMetS, mMetS: PLT↔ fMetS-DM, mMetS-DM: PLT↑	[71]

↑—increase, ↓—decrease, ↔—no changes, AHA—American Heart Association; BMI—body mass index; BP—blood pressure; CADP-CT—collagen and adenosine diphosphate closure time; CEPI-C—collagen and epinephrine closure time; DM2—type 2 diabetes mellitus; fMetS—female with metabolic syndrome; fMetS-DM—female with metabolic Syndrome and confirmed type 2 diabetes mellitus; MetS—metabolic syndrome; mMetS—male with metabolic syndrome; mMetS-DM—male with metabolic syndrome and type 2 diabetes mellitus; PLT—platelet count.

### 3.7. Impact of Chokeberry on Liver Functions

The liver has many important functions in the body. It is responsible for functions such as detoxification processes, production of coagulation factors, bile, storage of iron and some vitamins, as well as the metabolism of proteins, lipids, and carbohydrates [189]. Chronic liver diseases, including alcoholic liver disease and non-alcoholic fatty liver disease (NAFLD), hepatitis, liver fibrosis, cirrhosis, drug-induced liver diseases, and viral hepatitis, can lead to disruption of its functioning [190]. One of the symptoms is an increase in the level of liver enzymes, i.e., alanine aminotransferase (ALT), aspartate aminotransferase (AST), γ-glutamyl transferase (GGTP) [191]. An extremely important role in liver diseases is played by antioxidants that reduce inflammation and increase its detoxification functions [192,193]. The hepatoprotective effect of chokeberry has been shown in animal studies [25,26,27,28,194]. In one study on rats, the effect of a polyphenol-rich chokeberry extract on the modulation of microbiota was observed, which in turn improved the intestinal barrier function and at the same time the efficiency of the liver. The authors considered the influence of the main polyphenols of aronia, i.e., anthocyanidins, flavonols, and hydroxycinnamates [25]. In another rat study, a reduction in fatty liver disease in NAFLD was observed due to chokeberry extract inhibiting the expression of genes responsible for de novo lipogenesis in the liver, including sterol regulatory element-binding protein, acetyl-CoA carboxylase, and fatty acid synthase [27]. In a study on C57BL/6N mice fed a high-fat diet, inhibition expression of peroxisome proliferator-activated receptors (PPARs) improved liver functions, lipid profiles, and antioxidant capacity in response to treatments of chokeberry extract [195].

Yang et al. (2020) examined the anti-fibrotic effect of chokeberry anthocyanins on the liver of mice. Liver damage was caused by carbon tetrachloride. It has been shown to inhibit fibrosis by reducing the expression of proinflammatory factors, including interleukins and TNFα, inhibition of transforming growth factor β1 (TGF-β1) production and collagen I deposition and α-smooth muscle actin (α-SMA) expression [196]. Kozłowska et al. (2020) also confirmed the beneficial effect of chokeberry supplementation on the inhibition of liver fibrosis in rats exposed to cadmium by improving collagen protein homeostasis [197]. The strong antioxidant properties of chokeberry may also protect against exposure to heavy elements. Mężyńska et al. (2019) assessed the effect of 0.1% chokeberry extract on the liver of rats with low and moderate environmental exposure to cadmium. Administration of chokeberry extract decreased the concentration of prooxidants and oxidative stress in the liver, lowered the level of liver enzymes, and enhanced the action of protective enzymatic and non-enzymatic mechanisms in the liver [194].

In studies on humans, there are also positive indications of the effects of chokeberry. In a study by Tasic et al. (2021), they noted a decrease in AST in men and women with MetS without diabetes after 2 and 4 weeks of using chokeberry juice, while a decrease in AST in people with diabetes was only observed in women. A decrease in ALT was found in non-diabetic women with MetS after 4 weeks of consumption of chokeberry juice. A reduction was also observed in diabetic women after 2 weeks of using the juice, but after 4 weeks there was a slight increase in this enzyme. No effect was found in both non-diabetic and diabetic men with MetS. The effect on the surprising increase in direct bilirubin was observed after 4 weeks only in women and men with accompanying diabetes. The effect was not seen in people with MetS without diabetes [71]. However, no significant changes were found in a study by Kardum, Milovanović et al. (2015), in which chokeberry juice was consumed by people with the presence of high normal BP or grade I hypertension [79]. Gancheva et al. (2021) observed the effect of chokeberry juice only on the level of GGTP, i.e., a decrease from 33.9 to 29.3 U/L after 3 months, which was not observed by Loo et al. (2016), where GGTP levels remained unchanged after 2 months of intervention [76,104]. In the case of healthy individuals, in a study by Kardum, Konić-Ristić et al. (2014), no effect of supplementation with organic chokeberry juice (100 mL/day) on ALT and AST was observed [80].

Research shows that chokeberry may have a beneficial effect by increasing antioxidant potential, but no significant effect on the level of liver enzymes has been reported. Perhaps the consumption time should be longer, therefore, it seems that it requires high-quality clinical trials.

**Table 8:** Impact of chokeberry on liver functions in intervention studies.

Number of Participants (*n*) (Women/Men)	Characteristics of the Group	Type of Chokeberry Product	Dose of Chokeberry Product per Day	Time of Intervention (Weeks)	Changes in Diet	Results	References
*n* = 23 (11/12)	High normal BP or grade I hypertension: SBP = 130–159 mmHg, DBP = 85–99 mmHg, no regular use of antihypertensive drugs was declared by 23 patients (mean age 47.5, BMI nd)	Organic chokeberry juice (Conimex Trade d.o.o., Belgrade, Serbia)	200 mL	4	No changes	AST↔ ALT↔	[79]
*n* = 38 (24/14)	Mildly elevated BP: SBP 130–159 mmHg, DBP: 85–99 mmHg (mean age 55.8 years, BMI < 35) without DM2	Cold-pressed 100% chokeberry juice (Kiantama Ltd, Finland) or convection oven-dried chokeberry powder (Finnish Berry Powders Ltd., Finland)	300 mL chokeberry juice or 3 g dried chokeberry powder	8	No changes	GGTP↔	[76]
*n*= 22 (13/9)	overweight (*n* = 11, mean age 51.9, BMI 25–30 kg/m^2^), healthy (*n* = 11, mean age 41.4, BMI 18–25 kg/m^2^)	Chokeberry juice (Aronia Alive Agriculture Ltd.,Sofia, Bulgaria)	150 mL (50 mL, three times daily before meals)	12	nd	ALT↔ AST↔ GGTP↓	[104]
*n* = 144 (74/70)	MetS according to the AHA guidelines (age 50–60, BMI 30.1–34.4 kg/m^2^) I. *n* = 42, fMetS II. *n* = 42, mMetS III. *n* = 32, fMetS-DM IV. *n* = 28, mMetS-DM	standarized chokeberry extract (Alixir 400 PROTECT, Pharmanova, Belgrade, Serbia)	30 mL (prior or during dinner)	4	No changes	fMetS: AST↓, ALT↓, dBIL↔ mMetS: AST↓, ALT↔, dBIL↔ fMetS-DM: AST↓, ALT↔, dBIL↑ mMetS-DM: AST↔, ALT↔, dBIL↑	[71]

↑—increase, ↓—decrease, ↔—no changes, AHA—American Heart Association; ALT—alanine transaminase; AST—aspartate transaminase; BMI—body mass index; BP—blood pressure; dBIL—direct bilirubin; DM2—type 2 diabetes mellitus; fMetS—female with metabolic syndrome; fMetS-DM—female with metabolic syndrome and confirmed type 2 diabetes mellitus; GGTP—gamma-glutamyl transpeptidase; MetS—metabolic syndrome; mMetS—male with metabolic syndrome; mMetS-DM—male with metabolic syndrome and type 2 diabetes mellitus; nd—no data.

### 3.8. Impact Chokeberry on Uric Acid and Creatinine Level

Uric acid is a product of the enzymatic breakdown of purine nucleosides and free nitrogen bases [198]. Elevated levels of uric acid are observed, among other things, in obesity, hypertriglyceridemia, hypertension, kidney diseases, and diabetes [199,200]. Studies have also shown a relationship between hyperuricemia and the components of MetS [201,202,203,204]. However, its antioxidant and pro-oxidative activity in the abovementioned diseases is taken into account [205,206].

A slight increase in uric acid was observed after 3 months of consuming chokeberry juice by healthy people in a study by Kardum, Konić-Ristić et al. (2014), however, this increase was not statistically significant [80]. The effect was also not visible in people with MetS [77], hypercholesterolemia [103], and in people with the presence of high normal BP or grade I hypertension [79]. As shown in a study from 2021 carried out on mice, chokeberry anthocyanins may be beneficial in renal ischemia-reperfusion injury in mice. Mice with kidney damage were given a mixture of cyanidins (cyanidin-3-arabinoside, cyanidin-3-glucoside, and cyanidin-3-galactoside) and anthocyanins at 50 mg/g/12 h. Administration of the mixture resulted in an improvement in kidney function by alleviating the degree of kidney damage, and reducing proinflammatory cytokines, oxidative stress, creatinine levels, apoptosis, and lipid peroxidation [207]. The level of creatinine, in turn, can be a sign of the condition of the kidneys. However, it is emphasized that it is not the best diagnostic indicator due to its variability. Creatinine values may fluctuate depending on the level of muscle mass, dietary protein intake, or medications used [208]. In a study by Milutinović et al. (2019), creatinine in the blood decreased after 3 months, but it was not statistically significant. Moreover, after 6 months of supplementation, there was a significant increase in creatinine as compared with the value after 3 months of supplementation. Creatinine values determined in urine fell by about 32% after 3 months, while after the next 3 months, the level was similar to the value from before the intervention [100]. In a study by Kardum, Milovanović et al. (2015), blood creatinine remained unchanged [79]. The impact chokeberry on creatinine level remains unclear.

Another indication of the condition of kidneys is microalbuminuria. Microalbuminuria is the appearance of tiny amounts of protein in the urine. It is used as a diagnostic indicator of kidney disease, especially in diabetes. However, its high level may also be associated with a higher risk of heart disease [209]. A meta-analysis from 2014 showed a correlation between a decrease in the level of albumins in the urine and a decrease in cardiovascular events in patients with hypertension and/or diabetes [210]. In a meta-analysis from 2021, it was shown that microalbuminuria defined as urinary albumin excretion (30–300 mg/24 h urine) or albumin/creatinine ratio 30–300 mg/g from a spot urine or equivalent value, showed an independent relationship with all-cause mortality or major adverse cardiovascular events in hypertensive patients [211]. In a study by Milutinović et al. (2019), there were no statistically significant changes in the level of albumin in the urine after a 3-month intervention with chokeberry juice [100].

It follows that chokeberry does not affect uric acid levels, however, as mentioned earlier, it may be related to a short intervention time. The effects on kidney health also require further research due to a lack of research in this area.

**Table 9:** Impact of chokeberry on uric acid and creatinine levels in intervention studies.

Number of Participants (*n*) (Women/Men)	Characteristics of the Group	Type of Chokeberry Product	Dose of Chokeberry Product per Day	Time of Intervention (Weeks)	Changes in Diet	Results	References
*n* = 58 (0/58)	Mild hypocholesterolemia (TC > 200 mg/dL) without pharmacological treatment (mean age 54.1, BMI 27.7 kg/m^2^)	Organic chokebery juice (A. M. Lech Dzieciolowo, Poland)	250 mL	18 (12 weeks with drinking chokeberry juice)	No changes	uric acid↔	[103]
*n* = 47 (32/15)	MetS (*n* = 25, age 42–65, BMI 31.05 kg/m^2^), healthy (*n* = 22, BMI 24.15 kg/m^2^)	Chokeberry extract (Agropharm, Poland)	3 × 100 mg	8	No changes	uric acid↔	[77]
*n* = 23 (11/12)	High normal BP or grade I hypertension: SBP = 130–159 mmHg, DBP = 85–99 mmHg, no regular use of antihypertensive drugs (mean age 47.5, BMI nd)	Organic chokeberry juice (Conimex Trade d.o.o., Belgrade, Serbia)	200 mL	4	No changes	uric acid↔, urea↔, creatinine ↔	[79]
*n* = 35 (23/12)	DM2 and oral antidiabetic drugs therapy for at least 6 months (mean age 56.3, BMI 28.8 kg/m^2^)	Chokeberry juice (Nutrica d.o.o., Belgrade, Serbia)	150 mL (three times daily for 50 mL)	12	No changes	creatinine↓, urea↔, urine creatinine↔, microalbuminuria↔	[100]

↑—increase, ↓—decrease, ↔—no changes, BMI—body mass index; BP—blood pressure; DBP—diastolic blood pressure; DM2—type 2 diabetes mellitus; MetS—metabolic syndrome; nd—no data; SBP—systolic blood pressure.

The strength of our literature review is the fact that it comprehensively presents various directions of the effects of chokeberry fruit on metabolic disorders, and it collects and clearly summarizes scientific evidence for the effectiveness of chokeberry fruit. The review also gives recommendations as to the use of specific amounts of chokeberry juice or extract in particular metabolic disorders. Additionally, the review of the literature revealed shortcomings in the research carried out so far, which gives an indication of what future research should be directed at.

This literature review has several limitations. Manuscripts published after 2000 were reviewed, perhaps earlier work could cover other aspects that were not described in later publications. However, we wanted to present more recent scientific reports. Moreover, despite a careful literature review, it is possible that some studies may have been omitted. Additionally, most of the studies were conducted in the European population, which may cause bias in the interpretation of the results. It is also worth adding that metabolic disorders are usually interrelated and are not one separate disorder which translates into difficulties in interpreting the results.

## 4. Conclusions

Nowadays, we can observe the common occurrence of many metabolic disorders. Chokeberry products, especially juice and extract, are still widely researched. According to the review, chokeberry may have beneficial effects on certain disorders because it is high in antioxidants. A positive effect on glucose and lipid metabolism, blood pressure, and increasing the antioxidant potential were observed. A positive effect on carbohydrate metabolism was observed when consuming chokeberry juice for a minimum of 10 weeks (150–300 mL juice daily). In turn, for a standardized extract, the hypoglycemic effect in people with diabetes may occur after 4 weeks. The amount of juice that can influence the pressure varies according to the time of consumption. The hypotensive effect may occur after 4–12 weeks (200–300 mL of juice daily). In turn, for the extract, this time ranges from 4 to 6 weeks. The effect on LDL cholesterol was observed after 12 weeks (150 mL of juice per day), and in the case of the extract after 4 weeks. Triglycerides can decrease even after 4 weeks of consumption of the juice (200 mL per day). The influence on the other metabolic parameters discussed in this paper is not clear. However, animal studies assessing the effects of chokeberry on liver function, blood clotting, and kidney function are promising, whilemore human studies are required. Chokeberry is a product with high nutritional value, therefore, it can be recommended as part of the daily diet, for example, in the form of juice, for healthy and sick people. However, further clinical trials are needed to draw firm conclusions about its effects on other disorders.

## Data Availability

Not applicable.

## References

[1] Navab M., Gharavi N., Watson A.D. (2008). Inflammation and metabolic disorders. Curr. Opin. Clin. Nutr. Metab. Care.

[2] Hotamisligil G.S. (2006). Inflammation and metabolic disorders. Nature.

[3] Kopp W. (2019). How Western Diet And Lifestyle Drive The Pandemic Of Obesity And Civilization Diseases. Diabetes, Metab. Syndr. Obes. Targets Ther..

[4] Zorena K., Jachimowicz-Duda O., Ślęzak D., Robakowska M., Mrugacz M. (2020). Adipokines and Obesity. Potential Link to Metabolic Disorders and Chronic Complications. Int. J. Mol. Sci..

[5] World Health Organization Body Mass Index-BMI. https://www.euro.who.int/en/health-topics/disease-prevention/nutrition/a-healthy-lifestyle/body-mass-index-bmi.

[6] Ronti T., Lupattelli G., Mannarino E. (2006). The endocrine function of adipose tissue: An update. Clin. Endocrinol..

[7] Rani V., Deep G., Singh R.K., Palle K., Yadav U.C.S. (2016). Oxidative stress and metabolic disorders: Pathogenesis and therapeutic strategies. Life Sci..

[8] Reaven G.M. (1988). Banting lecture 1988. Role of insulin resistance in human disease. Diabetes.

[9] Oda E. (2012). Metabolic syndrome: Its history, mechanisms, and limitations. Acta Diabetol..

[10] Grundy S.M. (2015). Metabolic syndrome update. Trends Cardiovasc. Med..

[11] International Diabetes Federation The IDF Consensus Worldwide Definition of the Metabolic Syndrome. https://www.idf.org/e-library/consensus-statements/60-idfconsensus-worldwide-definitionof-the-metabolic-syndrome.html.

[12] Grundy S.M., Cleeman J.I., Daniels S.R., Donato K.A., Eckel R.H., Franklin B.A., Gordon D.J., Krauss R.M., Savage P.J., Smith S.C. (2005). Diagnosis and management of the metabolic syndrome: An American Heart Association/National Heart, Lung, and Blood Institute scientific statement. Circulation.

[13] Alberti K.G.M.M., Eckel R.H., Grundy S.M., Zimmet P.Z., Cleeman J.I., Donato K.A., Fruchart J.C., James W.P.T., Loria C.M., Smith S.C. (2009). Harmonizing the metabolic syndrome: A joint interim statement of the international diabetes federation task force on epidemiology and prevention; National heart, lung, and blood institute; American heart association; World heart federation; International Atherosclerosis Society; and International Association for the Study of Obesity. Circulation.

[14] Gurka M.J., Filipp S.L., DeBoer M.D. (2018). Geographical variation in the prevalence of obesity, metabolic syndrome, and diabetes among US adults. Nutr. Diabetes.

[15] Scuteri A., Laurent S., Cucca F., Cockcroft J., Cunha P., Rodríguez-Mañas L., Raso F.U.M., Muiesan M.L., Ryliškytė L., Rietzschel E. (2015). Metabolic syndrome across Europe: Different clusters of risk factors. Eur. J. Prev. Cardiol..

[16] Zujko M.E., Waśkiewicz A., Witkowska A.M., Szcześniewska D., Zdrojewski T., Kozakiewicz K., Drygas W. (2018). Dietary Total Antioxidant Capacity and Dietary Polyphenol Intake and Prevalence of Metabolic Syndrome in Polish Adults: A Nationwide Study. Oxidative Med. Cell. Longev..

[17] Gregório B.M., De Souza D.B., Nascimento F.A.D.M., Matta L., Fernandes-Santos C. (2016). The Potential Role of Antioxidants in Metabolic Syndrome. Curr. Pharm. Des..

[18] Soory M. (2009). Relevance of nutritional antioxidants in metabolic syndrome, ageing and cancer: Potential for therapeutic targeting. Infect. Disord. Drug Targets.

[19] Tolić M.-T., Jurčević I.L., Krbavčić I.P., Marković K., Vahčić N. (2015). Phenolic Content, Antioxidant Capacity and Quality of Chokeberry (*Aronia melanocarpa*) Products. Food Technol. Biotechnol..

[20] Jakobek L., Šeruga M., Krivak P. (2011). The influence of interactions among phenolic compounds on the antiradical activity of chokeberries (*Aronia melanocarpa*). Int. J. Food Sci. Nutr..

[21] Rop O., Mlcek J., Jurikova T., Valsikova M., Sochor J., Reznicek V., Kramarova D. (2010). Phenolic content, antioxidant capacity, radical oxygen species scavenging and lipid peroxidation inhibiting activities of extracts of five black chokeberry (*Aronia melanocarpa* (Michx.) Elliot) cultivars. J. Med. Plant Res..

[22] Jeon Y.-D., Kang S.-H., Moon K.-H., Lee J.-H., Kim D.-G., Kim W., Kim J.-S., Ahn B.-Y., Jin J.-S. (2018). The Effect of Aronia Berry on Type 1 Diabetes In Vivo and In Vitro. J. Med. Food.

[23] Takahashi A., Shimizu H., Okazaki Y., Sakaguchi H., Taira T., Suzuki T., Chiji H. (2015). Anthocyanin-rich Phytochemicals from Aronia Fruits Inhibit Visceral Fat Accumulation and Hyperglycemia in High-fat Diet-induced Dietary Obese Rats. J. Oleo Sci..

[24] Valcheva-Kuzmanova S., Kuzmanov K., Tancheva S., Belcheva A. (2007). Hypoglycemic effects of Aronia melanocarpa fruit juice in streptozotocin-induced diabetic rats. Methods Find. Exp. Clin. Pharmacol..

[25] Kong Y., Yan T., Tong Y., Deng H., Tan C., Wan M., Wang M., Meng X., Wang Y. (2021). Gut Microbiota Modulation by Polyphenols from *Aronia melanocarpa* of LPS-Induced Liver Diseases in Rats. J. Agric. Food Chem..

[26] Kondeva-Burdina M., Valcheva-Kuzmanova S., Markova T., Mitcheva M., Belcheva A. (2015). Effects of aronia melanocarpa fruit juice on isolated rat hepatocytes. Pharmacogn. Mag..

[27] Park H., Liu Y., Kim H.-S., Shin J.-H. (2016). Chokeberry attenuates the expression of genes related to de novo lipogenesis in the hepatocytes of mice with nonalcoholic fatty liver disease. Nutr. Res..

[28] Valcheva-Kuzmanova S., Borisova P., Galunska B., Krasnaliev I., Belcheva A. (2004). Hepatoprotective effect of the natural fruit juice from Aronia melanocarpa on carbon tetrachloride-induced acute liver damage in rats. Exp. Toxicol. Pathol..

[29] Piotrowska-Kempisty H., Nowicki M., Jodynis-Liebert J., Kurpik M., Ewertowska M., Adamska T., Oszmiański J., Kujawska M. (2020). Assessment of Hepatoprotective Effect of Chokeberry Juice in Rats Treated Chronically with Carbon Tetrachloride. Molecules.

[30] Bhaswant M., Shafie S.R., Mathai M.L., Mouatt P., Brown L. (2017). Anthocyanins in chokeberry and purple maize attenuate diet-induced metabolic syndrome in rats. Nutrition.

[31] Ciocoiu M., Badescu L., Miron A., Badescu M. (2013). The Involvement of a Polyphenol-Rich Extract of Black Chokeberry in Oxidative Stress on Experimental Arterial Hypertension. Evid.-Based Complement. Altern. Med..

[32] Banihani S., Swedan S., Alguraan Z. (2013). Pomegranate and type 2 diabetes. Nutr. Res..

[33] Lim S.-M., Lee H.S., Jung J.I., Kim S.M., Kim N.Y., Seo T.S., Bae J.-S., Kim E.J. (2019). Cyanidin-3-*O*-Galactoside-Enriched *Aronia melanocarpa* Extract Attenuates Weight Gain and Adipogenic Pathways in High-Fat Diet-Induced Obese C57BL/6 Mice. Nutrients.

[34] Daskalova E., Delchev S., Peeva Y., Vladimirova-Kitova L., Kratchanova M., Kratchanov C., Denev P. (2015). Antiatherogenic and Cardioprotective Effects of Black Chokeberry (*Aronia melanocarpa*) Juice in Aging Rats. Evid.-Based Complement. Altern. Med..

[35] Valcheva-Kuzmanova S., Kuzmanov K., Mihova V., Krasnaliev I., Borisova P., Belcheva A. (2007). Antihyperlipidemic Effect of Aronia melanocarpa Fruit Juice in Rats Fed a High-Cholesterol Diet. Plant Foods Hum. Nutr..

[36] Zhao Y., Liu X., Zheng Y., Liu W., Ding C. (2021). Aronia melanocarpa polysaccharide ameliorates inflammation and aging in mice by modulating the AMPK/SIRT1/NF-κB signaling pathway and gut microbiota. Sci. Rep..

[37] Shatoor A.S., Al Humayed S. (2020). The Protective Effect of Crataegus aronia Against High-Fat Diet-Induced Vascular Inflammation in Rats Entails Inhibition of the NLRP-3 Inflammasome Pathway. Cardiovasc. Toxicol..

[38] Yu S.-Y., Kim M.-B., Park Y.-K., Bae M., Kang H., Hu S., Pham T.X., Carpenter R., Lee J., Lee O.-H. (2021). Anthocyanin-Rich Aronia Berry Extract Mitigates High-Fat and High-Sucrose Diet-Induced Adipose Tissue Inflammation by Inhibiting Nuclear Factor-*κ*B Activation. J. Med. Food.

[39] Kang S.-H., Jeon Y.-D., Moon K.-H., Lee J.-H., Kim D.-G., Kim W., Myung H., Kim J.-S., Kim H.-J., Bang K.-S. (2017). Aronia Berry Extract Ameliorates the Severity of Dextran Sodium Sulfate-Induced Ulcerative Colitis in Mice. J. Med. Food.

[40] Valcheva-Kuzmanova S., Kuzmanov A., Kuzmanova V., Tzaneva M. (2018). Aronia melanocarpa fruit juice ameliorates the symptoms of inflammatory bowel disease in TNBS-induced colitis in rats. Food Chem. Toxicol..

[41] Pei R., Liu J., Martin D.A., Valdez J.C., Jeffety J., Barrett-Wilt G.A., Liu Z., Bolling B.W. (2019). Aronia Berry Supplementation Mitigates Inflammation in T Cell Transfer-Induced Colitis by Decreasing Oxidative Stress. Nutrients.

[42] Tomić M., Ignjatović Đ., Tovilović-Kovačević G., Krstić-Milošević D., Ranković S., Popović T., Glibetić M. (2016). Reduction of anxiety-like and depression-like behaviors in rats after one month of drinking Aronia melanocarpa berry juice. Food Funct..

[43] Wei J., Zhang G., Zhang X., Xu D., Gao J., Fan J., Zhou Z. (2017). Anthocyanins from Black Chokeberry (*Aroniamelanocarpa* Elliot) Delayed Aging-Related Degenerative Changes of Brain. J. Agric. Food Chem..

[44] Wen H., Cui H., Tian H., Zhang X., Ma L., Ramassamy C., Li J. (2020). Isolation of Neuroprotective Anthocyanins from Black Chokeberry (*Aronia melanocarpa*) against Amyloid-β-Induced Cognitive Impairment. Foods.

[45] Daskalova E., Delchev S., Topolov M., Dimitrova S., Uzunova Y., Valcheva-Kuzmanova S., Kratchanova M., Vladimirova-Kitova L., Denev P. (2019). *Aronia melanocarpa* (Michx.) Elliot fruit juice reveals neuroprotective effect and improves cognitive and locomotor functions of aged rats. Food Chem. Toxicol..

[46] Gajić D., Saksida T., Koprivica I., Šenerović L., Morić I., Šavikin K., Menković N., Pejnović N., Stojanović I. (2020). Immunomodulatory activity and protective effects of chokeberry fruit extract on Listeria monocytogenes infection in mice. Food Funct..

[47] Park S., Kim J.I., Lee I., Lee S., Hwang M.-W., Bae J.-Y., Heo J., Kim D., Han S.-Z., Park M.-S. (2013). Aronia melanocarpa and its components demonstrate antiviral activity against influenza viruses. Biochem. Biophys. Res. Commun..

[48] Hawkins J., Hires C., Baker C., Keenan L., Bush M. (2020). Daily supplementation with aronia melanocarpa (chokeberry) reduces blood pressure and cholesterol: A meta analysis of controlled clinical trials. J. Diet. Suppl..

[49] Rahmani J., Clark C., Varkaneh H.K., Lakiang T., Vasanthan L.T., Onyeche V., Mousavi S.M., Zhang Y. (2019). The effect of Aronia consumption on lipid profile, blood pressure, and biomarkers of inflammation: A systematic review and meta-analysis of randomized controlled trials. Phytother. Res..

[50] Zheng W., Wang S.Y. (2003). Oxygen Radical Absorbing Capacity of Phenolics in Blueberries, Cranberries, Chokeberries, and Lingonberries. J. Agric. Food Chem..

[51] Sidor A., Gramza-Michałowska A. (2019). Black Chokeberry *Aronia melanocarpa* L.—A Qualitative Composition, Phenolic Profile and Antioxidant Potential. Molecules.

[52] King E.S., Bolling B.W., Kiritsakis A.K., Kiritsakis K.A., Tsitsipas C.K. (2020). Composition, polyphenol bioavailability, and health benefits of aronia berry: A review. J. Food Bioact..

[53] Trenka M., Nawirska-Olszańska A., Oziembłowski M. (2020). Analysis of Selected Properties of Fruits of Black Chokeberry (*Aronia melanocarpa* L.) from Organic and Conventional Cultivation. Appl. Sci..

[54] Zielińska A., Siudem P., Paradowska K., Gralec M., Kaźmierski S., Wawer I. (2020). *Aronia melanocarpa* Fruits as a Rich Dietary Source of Chlorogenic Acids and Anthocyanins: ^1^H-NMR, HPLC-DAD, and Chemometric Studies. Molecules.

[55] Putta S., Yarla N.S., Kumar E.K., Lakkappa D.B., Kamal M.A., Scotti L., Scotti M.T., Ashraf G., Rao B.S.B., Kumari S. (2019). Preventive and Therapeutic Potentials of Anthocyanins in Diabetes and Associated Complications. Curr. Med. Chem..

[56] Oszmiański J., Lachowicz S. (2016). Effect of the Production of Dried Fruits and Juice from Chokeberry (*Aronia melanocarpa* L.) on the Content and Antioxidative Activity of Bioactive Compounds. Molecules.

[57] Guo H., Xia M., Watson R.R., Preedy V.R., Zibadi S. (2018). Chapter 12—Anthocyanins and Diabetes Regulation. Polyphenols: Mechanisms of Action in Human Health and Disease.

[58] Cindrić I.J., Zeiner M., Mihajlov-Konanov D., Stingeder G. (2017). Inorganic Macro- and Micronutrients in “Superberries” Black Chokeberries (*Aronia melanocarpa*) and Related Teas. Int. J. Environ. Res. Public Health.

[59] Rodríguez-Werner M., Winterhalter P., Esatbeyoglu T. (2019). Phenolic Composition, Radical Scavenging Activity and an Approach for Authentication of *Aronia melanocarpa* Berries, Juice, and Pomace. J. Food Sci..

[60] Romani A., Vignolini P., Ieri F., Heimler D. (2016). Polyphenols and Volatile Compounds in Commercial Chokeberry (*Aronia melanocarpa*) Products. Nat. Prod. Commun..

[61] Bolling B.W., Taheri R., Pei R., Kranz S., Yu M., Durocher S.N., Brand M.H. (2015). Harvest date affects aronia juice polyphenols, sugars, and antioxidant activity, but not anthocyanin stability. Food Chem..

[62] Wilkes K., Howard L.R., Brownmiller C., Prior R.L. (2014). Changes in Chokeberry (*Aronia melanocarpa* L.) Polyphenols during Juice Processing and Storage. J. Agric. Food Chem..

[63] Hwang E.-S., Thi N.D. (2016). Effects of Different Growing Regions on Quality Characteristics, Bioactive Compound Contents, and Antioxidant Activity of Aronia (*Aronia melanocarpa*) in Korea. Prev. Nutr. Food Sci..

[64] Casadei K., Kiel J. (2022). Anthropometric Measurement. StatPearls.

[65] Cetin D., Lessig B.A., Nasr E. (2016). Comprehensive Evaluation for Obesity: Beyond Body Mass Index. J. Osteopat. Med..

[66] Wang S., Moustaid-Moussa N., Chen L., Mo H., Shastri A., Su R., Bapat P., Kwun I., Shen C.-L. (2014). Novel insights of dietary polyphenols and obesity. J. Nutr. Biochem..

[67] Akhlaghi M., Ghobadi S., Hosseini M.M., Gholami Z., Mohammadian F. (2018). Flavanols are potential anti-obesity agents, a systematic review and meta-analysis of controlled clinical trials. Nutr. Metab. Cardiovasc. Dis..

[68] Prior R.L., Wu X., Gu L., Hager T.J., Hager A., Howard L.R. (2008). Whole Berries versus Berry Anthocyanins: Interactions with Dietary Fat Levels in the C57BL/6J Mouse Model of Obesity. J. Agric. Food Chem..

[69] Kim N.-Y., Thomas S.S., Hwang D.-I., Lee J.-H., Kim K.-A., Cha Y.-S. (2021). Anti-Obesity Effects of *Morus alba* L. and *Aronia melanocarpa* in a High-Fat Diet-Induced Obese C57BL/6J Mouse Model. Foods.

[70] Kowalska K., Olejnik A., Szwajgier D., Olkowicz M. (2017). Inhibitory activity of chokeberry, bilberry, raspberry and cranberry polyphenol-rich extract towards adipogenesis and oxidative stress in differentiated 3T3-L1 adipose cells. PLoS ONE.

[71] Tasic N., Jakovljevic V.L.J., Mitrovic M., Djindjic B., Tasic D., Dragisic D., Citakovic Z., Kovacevic Z., Radoman K., Zivkovic V. (2021). Black chokeberry Aronia melanocarpa extract reduces blood pressure, glycemia and lipid profile in patients with metabolic syndrome: A prospective controlled trial. Mol. Cell. Biochem..

[72] Kardum N., Petrović-Oggiano G., Takic M., Glibetić N., Zec M., Debeljak-Martacic J., Konić-Ristić A. (2014). Effects of Glucomannan-Enriched, Aronia Juice-Based Supplement on Cellular Antioxidant Enzymes and Membrane Lipid Status in Subjects with Abdominal Obesity. Sci. World J..

[73] Pokimica B., García-Conesa M.-T., Zec M., Debeljak-Martačić J., Ranković S., Vidović N., Petrović-Oggiano G., Konić-Ristić A., Glibetić M. (2019). Chokeberry Juice Containing Polyphenols Does Not Affect Cholesterol or Blood Pressure but Modifies the Composition of Plasma Phospholipids Fatty Acids in Individuals at Cardiovascular Risk. Nutrients.

[74] Sikora J., Broncel M., Markowicz M., Chałubiński M., Wojdan K., Mikiciuk-Olasik E. (2012). Short-term supplementation with Aronia melanocarpa extract improves platelet aggregation, clotting, and fibrinolysis in patients with metabolic syndrome. Eur. J. Nutr..

[75] Sikora J., Broncel M., Mikiciuk-Olasik E. (2014). Aronia melanocarpa ElliotReduces the Activity of Angiotensin I-Converting Enzyme—In Vitro and Ex Vivo Studies. Oxidative Med. Cell. Longev..

[76] Loo B.-M., Erlund I., Koli R., Puukka P., Hellström J., Wähälä K., Mattila P., Jula A. (2016). Consumption of chokeberry (*Aronia mitschurinii*) products modestly lowered blood pressure and reduced low-grade inflammation in patients with mildly elevated blood pressure. Nutr. Res..

[77] Broncel M., Kozirog M., Duchnowicz P., Koter-Michalak M., Sikora J., Chojnowska-Jezierska J. (2010). Aronia melanocarpa extract reduces blood pressure, serum endothelin, lipid, and oxidative stress marker levels in patients with metabolic syndrome. Med. Sci. Monit..

[78] Naruszewicz M., Łaniewska I., Millo B., Dłużniewski M. (2007). Combination therapy of statin with flavonoids rich extract from chokeberry fruits enhanced reduction in cardiovascular risk markers in patients after myocardial infraction (MI). Atherosclerosis.

[79] Kardum N., Milovanović B., Šavikin K., Zdunić G., Mutavdžin S., Gligorijević T., Spasić S. (2015). Beneficial Effects of Polyphenol-Rich Chokeberry Juice Consumption on Blood Pressure Level and Lipid Status in Hypertensive Subjects. J. Med. Food.

[80] Kardum N., Ristić A.K., Šavikin K., Spasić S., Stefanović A., Ivanišević J., Miljković M. (2014). Effects of Polyphenol-Rich Chokeberry Juice on Antioxidant/Pro-Oxidant Status in Healthy Subjects. J. Med. Food.

[81] Jebur A.B., Mokhamer M.H., El-Demerdash F.M. (2016). A Review on Oxidative Stress and Role of Antioxidants in Diabetes Mellitus. Austin Endocrinol. Diabetes Case Rep..

[82] Aryaeian N., Sedehi S.K., Arablou T. (2017). Polyphenols and their effects on diabetes management: A review. Med. J. Islam. Repub. Iran.

[83] Guasch-Ferré M., Merino J., Sun Q., Fitó M., Salas-Salvadó J. (2017). Dietary Polyphenols, Mediterranean Diet, Prediabetes, and Type 2 Diabetes: A Narrative Review of the Evidence. Oxid. Med. Cell. Longev..

[84] Schulze C., Bangert A., Kottra G., Geillinger K.E., Schwanck B., Vollert H., Blaschek W., Daniel H. (2014). Inhibition of the intestinal sodium-coupled glucose transporter 1 (SGLT1) by extracts and polyphenols from apple reduces postprandial blood glucose levels in mice and humans. Mol. Nutr. Food Res..

[85] Papuc C., Goran G.V., Predescu C.N., Tudoreanu L., Ștefan G. (2022). Plant polyphenols mechanisms of action on insulin resistance and against the loss of pancreatic beta cells. Crit. Rev. Food Sci. Nutr..

[86] Williamson G., Sheedy K. (2020). Effects of Polyphenols on Insulin Resistance. Nutrients.

[87] Toupchian O., Abdollahi S., Salehi-Abargouei A., Heshmati J., Clark C.C.T., Sheikhha M.H., Fallahzadeh H., Mozaffari-Khosravi H. (2021). The effects of resveratrol supplementation on *PPARα*, *p16*, *p53*, *p21* gene expressions, and sCD163/sTWEAK ratio in patients with type 2 diabetes mellitus: A double-blind controlled randomized trial. Phytother. Res..

[88] Yang L., Ling W., Yang Y., Chen Y., Tian Z., Du Z., Chen J., Xie Y., Liu Z., Yang L. (2017). Role of Purified Anthocyanins in Improving Cardiometabolic Risk Factors in Chinese Men and Women with Prediabetes or Early Untreated Diabetes—A Randomized Controlled Trial. Nutrients.

[89] Li D., Zhang Y., Liu Y., Sun R., Xia M. (2015). Purified Anthocyanin Supplementation Reduces Dyslipidemia, Enhances Antioxidant Capacity, and Prevents Insulin Resistance in Diabetic Patients. J. Nutr..

[90] Shehzad A., Iqbal W., Shehzad O., Lee Y.S. (2012). Adiponectin: Regulation of its production and its role in human diseases. Hormones.

[91] Nigro E., Scudiero O., Monaco M.L., Palmieri A., Mazzarella G., Costagliola C., Bianco A., Daniele A. (2014). New Insight into Adiponectin Role in Obesity and Obesity-Related Diseases. BioMed. Res. Int..

[92] Rugină D., Diaconeasa Z., Coman C., Bunea A., Socaciu C., Pintea A. (2015). Chokeberry Anthocyanin Extract as Pancreatic*β*-Cell Protectors in Two Models of Induced Oxidative Stress. Oxidative Med. Cell. Longev..

[93] Yamane T., Kozuka M., Konda D., Nakano Y., Nakagaki T., Ohkubo I., Ariga H. (2016). Improvement of blood glucose levels and obesity in mice given aronia juice by inhibition of dipeptidyl peptidase IV and α-glucosidase. J. Nutr. Biochem..

[94] Bräunlich M., Slimestad R., Wangensteen H., Brede C., Malterud K.E., Barsett H. (2013). Extracts, Anthocyanins and Procyanidins from Aronia melanocarpa as Radical Scavengers and Enzyme Inhibitors. Nutrients.

[95] DE S., Banerjee S., Kumar S.A., Paira P. (2019). Critical Role of Dipeptidyl Peptidase IV: A Therapeutic Target for Diabetes and Cancer. Mini-Rev. Med. Chem..

[96] Deacon C.F., Lebovitz H.E. (2016). Comparative review of dipeptidyl peptidase-4 inhibitors and sulphonylureas. Diabetes Obes. Metab..

[97] Kozuka M., Yamane T., Nakano Y., Nakagaki T., Ohkubo I., Ariga H. (2015). Identification and characterization of a dipeptidyl peptidase IV inhibitor from aronia juice. Biochem. Biophys. Res. Commun..

[98] Worsztynowicz P., Napierała M., Białas W., Grajek W., Olkowicz M. (2014). Pancreatic α-amylase and lipase inhibitory activity of polyphenolic compounds present in the extract of black chokeberry (*Aronia melanocarpa* L.). Process Biochem..

[99] Mu J., Xin G., Zhang B., Wang Y., Ning C., Meng X. (2020). Beneficial effects of *Aronia melanocarpa* berry extract on hepatic insulin resistance in type 2 diabetes mellitus rats. J. Food Sci..

[100] Milutinović M., Radovanović R.V., Šavikin K., Radenković S., Arvandi M., Pešić M., Kostić M., Miladinović B., Branković S., Kitić D. (2019). Chokeberry juice supplementation in type 2 diabetic patients—Impact on health status. J. Appl. Biomed..

[101] Nathan D.M., Turgeon H., Regan S. (2007). Relationship between glycated haemoglobin levels and mean glucose levels over time. Diabetologia.

[102] Lancrajan I. (2012). Aronia Melanocarpa, a Potential Therapeutic Agent. Studia Universitatis “Vasile Goldiş”. Ser. Ştiinţele Vieţii.

[103] Skoczyńska A., Jedrychowska I., Poręba R., Affelska-Jercha A., Turczyn B., Wojakowska A., Andrzejak R. (2007). Influence of chokeberry juice on arterial blood pressure and lipid parameters in men with mild hypercholesterolemia. Pharmacol. Rep..

[104] Gancheva S., Ivanova I., Atanassova A., Gancheva-Tomova D., Eftimov M., Moneva K., Zhelyazkova-Savova M., Valcheva-Kuzmanova S. (2021). Effects of Aronia melanocarpa fruit juice on oxidative stress, energy homeostasis, and liver function in overweight and healthy-weight individuals. Scr. Sci. Med..

[105] Yamane T., Kozuka M., Wada-Yoneta M., Sakamoto T., Nakagaki T., Nakano Y., Ohkubo I. (2017). Aronia juice suppresses the elevation of postprandial blood glucose levels in adult healthy Japanese. Clin. Nutr. Exp..

[106] Petrovic S., Arsic A., Glibetic M., Cikiriz N., Jakovljevic V., Vucic V. (2016). The effects of polyphenol-rich chokeberry juice on fatty acid profiles and lipid peroxidation of active handball players: Results from a randomized, double-blind, placebo-controlled study. Can. J. Physiol. Pharmacol..

[107] Kardum N., Takić M., Šavikin K., Zec M., Zdunić G., Spasić S., Konić-Ristić A. (2014). Effects of polyphenol-rich chokeberry juice on cellular antioxidant enzymes and membrane lipid status in healthy women. J. Funct. Foods.

[108] Unger T., Borghi C., Charchar F., Khan N.A., Poulter N.R., Prabhakaran D., Ramirez A., Schlaich M., Stergiou G.S., Tomaszewski M. (2020). 2020 International Society of Hypertension Global Hypertension Practice Guidelines. Hypertension.

[109] Kjeldsen S.E. (2018). Hypertension and cardiovascular risk: General aspects. Pharmacol. Res..

[110] Mills K.T., Stefanescu A., He J. (2020). The global epidemiology of hypertension. Nat. Rev. Nephrol..

[111] Ozemek C., Laddu D.R., Arena R., Lavie C.J. (2018). The role of diet for prevention and management of hypertension. Curr. Opin. Cardiol..

[112] Galleano M., Pechanova O., Fraga C.G. (2010). Hypertension, Nitric Oxide, Oxidants, and Dietary Plant Polyphenols. Curr. Pharm. Biotechnol..

[113] Godos J., Vitale M., Micek A., Ray S., Martini D., Del Rio D., Riccardi G., Galvano F., Grosso G. (2019). Dietary Polyphenol Intake, Blood Pressure, and Hypertension: A Systematic Review and Meta-Analysis of Observational Studies. Antioxidants.

[114] Hügel H.M., Jackson N., May B., Zhang A.L., Xue C.C. (2016). Polyphenol protection and treatment of hypertension. Phytomedicine.

[115] Vendrame S., Klimis-Zacas D. (2019). Potential Factors Influencing the Effects of Anthocyanins on Blood Pressure Regulation in Humans: A Review. Nutrients.

[116] Pavlovic A.N., Brcanović J.M., Veljković J.N., Mitic S.S., Tošić S., Kaličanin B.M., Kostic D.A., Ðorđević M.S., Velimirović D.S. (2015). Characterization of commercially available products of aronia according to their metal content. Fruits.

[117] Tjelle T.E., Holtung L., Bøhn S.K., Aaby K., Thoresen M., Wiik S., Paur I., Karlsen A.S., Retterstøl K., Iversen P.O. (2015). Polyphenol-rich juices reduce blood pressure measures in a randomised controlled trial in high normal and hypertensive volunteers. Br. J. Nutr..

[118] Ahles S., Stevens Y.R., Joris P.J., Vauzour D., Adam J., De Groot E., Plat J. (2020). The Effect of Long-Term *Aronia melanocarpa* Extract Supplementation on Cognitive Performance, Mood, and Vascular Function: A Randomized Controlled Trial in Healthy, Middle-Aged Individuals. Nutrients.

[119] Masuyer G., Yates C.J., Sturrock E.D., Acharya K.R. (2014). Angiotensin-I converting enzyme (ACE): Structure, biological roles, and molecular basis for chloride ion dependence. Biol. Chem..

[120] Piepho R.W. (2000). Overview of the angiotensin-converting-enzyme inhibitors. Am. J. Health Syst. Pharm..

[121] Shihoya W., Nishizawa T., Okuta A., Tani K., Dohmae N., Fujiyoshi W.S.A.O.K.T.Y., Nureki W.S.T.N.O., Doi T. (2016). Activation mechanism of endothelin ETB receptor by endothelin-1. Nature.

[122] Khimji A.-K., Rockey D.C. (2010). Endothelin—Biology and disease. Cell. Signal..

[123] Mannu G.S., Zaman M.J., Gupta A., Rehman H.U., Myint P.K. (2013). Evidence of Lifestyle Modification in the Management of Hypercholesterolemia. Curr. Cardiol. Rev..

[124] Sonestedt E., Hellstrand S., Drake I., Schulz C.-A., Ericson U., Hlebowicz J., Persson M.M., Gullberg B., Hedblad B., Engström G. (2016). Diet Quality and Change in Blood Lipids during 16 Years of Follow-up and Their Interaction with Genetic Risk for Dyslipidemia. Nutrients.

[125] Orozco-Beltran D., Gil-Guillen V.F., Redon J., Martin-Moreno J.M., Pallares-Carratala V., Navarro-Perez J., Valls-Roca F., Sanchis-Domenech C., Fernandez-Gimenez A., Perez-Navarro A. (2018). Correction: Lipid profile, cardiovascular disease and mortality in a Mediterranean high-risk population: The ESCARVAL-RISK study. PLoS ONE.

[126] Buchholz T., Melzig M.F. (2015). Polyphenolic Compounds as Pancreatic Lipase Inhibitors. Planta Med..

[127] Bialecka-Florjanczyk E., Fabiszewska A.U., Krzyczkowska J., Kurylowicz A. (2018). Synthetic and Natural Lipase Inhibitors. Mini-Reviews Med. Chem..

[128] Shishikura Y., Khokhar A.S., Murray B.S. (2006). Effects of Tea Polyphenols on Emulsification of Olive Oil in a Small Intestine Model System. J. Agric. Food Chem..

[129] Tung W.-C., Rizzo B., Dabbagh Y., Saraswat S., Romanczyk M., Codorniu-Hernández E., Rebollido-Rios R., Needs P.W., Kroon P.A., Rakotomanomana N. (2020). Polyphenols bind to low density lipoprotein at biologically relevant concentrations that are protective for heart disease. Arch. Biochem. Biophys..

[130] Guo H., Liu G., Zhong R., Wang Y., Wang D., Xia M. (2012). Cyanidin-3-O-β-glucoside regulates fatty acid metabolism via an AMP-activated protein kinase-dependent signaling pathway in human HepG2 cells. Lipids Health Dis..

[131] Mahdavi A., Bagherniya M., Fakheran O., Reiner Ž., Xu S., Sahebkar A. (2020). Medicinal plants and bioactive natural compounds as inhibitors of HMG-CoA reductase: A literature review. BioFactors.

[132] Towler M.C., Hardie G. (2007). AMP-Activated Protein Kinase in Metabolic Control and Insulin Signaling. Circ. Res..

[133] Lorrain B., Dangles O., Genot C., Dufour C. (2010). Chemical Modeling of Heme-Induced Lipid Oxidation in Gastric Conditions and Inhibition by Dietary Polyphenols. J. Agric. Food Chem..

[134] Lorrain B., Dangles O., Loonis M., Armand M., Dufour C. (2012). Dietary Iron-Initiated Lipid Oxidation and Its Inhibition by Polyphenols in Gastric Conditions. J. Agric. Food Chem..

[135] Kim B., Park Y., Wegner C.J., Bolling B.W., Lee J. (2013). Polyphenol-rich black chokeberry (*Aronia melanocarpa*) extract regulates the expression of genes critical for intestinal cholesterol flux in Caco-2 cells. J. Nutr. Biochem..

[136] Kim B., Ku C.S., Pham T.X., Park Y., Martin D.A., Xie L., Taheri R., Lee J., Bolling B. (2013). *Aronia melanocarpa* (chokeberry) polyphenol–rich extract improves antioxidant function and reduces total plasma cholesterol in apolipoprotein E knockout mice. Nutr. Res..

[137] Duchnowicz P., Ziobro A., Rapacka E., Koter-Michalak M., Bukowska B. (2018). Changes in Cholinesterase Activity in Blood of Adolescent with Metabolic Syndrome after Supplementation with Extract from *Aronia melanocarpa*. BioMed. Res. Int..

[138] Škovierová H., Vidomanová E., Mahmood S., Sopková J., Drgová A., Červeňová T., Halašová E., Lehotský J. (2016). The Molecular and Cellular Effect of Homocysteine Metabolism Imbalance on Human Health. Int. J. Mol. Sci..

[139] Lei X., Zeng G., Zhang Y., Li Q., Zhang J., Bai Z., Yang K. (2018). Association between homocysteine level and the risk of diabetic retinopathy: A systematic review and meta-analysis. Diabetol. Metab. Syndr..

[140] Agoston-Coldea L., Mocan T., Gatfosse M., Lupu S., Dumitrascu D.L. (2011). Plasma homocysteine and the severity of heart failure in patients with previous myocardial infarction. Cardiol. J..

[141] Yeh J.-K., Chen C.-C., Hsieh M.-J., Tsai M.-L., Yang C.-H., Chen D.-Y., Chang S.-H., Wang C.-Y., Lee C.-H., Hsieh I.-C. (2017). Impact of Homocysteine Level on Long-term Cardiovascular Outcomes in Patients after Coronary Artery Stenting. J. Atheroscler. Thromb..

[142] Hu Y., Hu F.B., Manson J.E. (2019). Marine Omega-3 Supplementation and Cardiovascular Disease: An Updated Meta-Analysis of 13 Randomized Controlled Trials Involving 127 477 Participants. J. Am. Heart Assoc..

[143] Kaneva A.M., Potolitsyna N.N., Bojko E.R., Odland J. (2015). The Apolipoprotein B/Apolipoprotein A-I Ratio as a Potential Marker of Plasma Atherogenicity. Dis. Markers.

[144] Xie L., Vance T., Kim B., Gil Lee S.G., Caceres C., Wang Y., Hubert P.A., Lee J.-Y., Chun O.K., Bolling B.W. (2016). Aronia berry polyphenol consumption reduces plasma total and low-density lipoprotein cholesterol in former smokers without lowering biomarkers of inflammation and oxidative stress: A randomized controlled trial. Nutr. Res..

[145] Nowak D., Grąbczewska Z., Gośliński M., Obońska K., Dąbrowska A., Kubica J. (2016). Effect of chokeberry juice consumption on antioxidant capacity, lipids profile and endothelial function in healthy people: A pilot study. Czech J. Food Sci..

[146] Di Gioia M., Zanoni I. (2021). Dooming Phagocyte Responses: Inflammatory Effects of Endogenous Oxidized Phospholipids. Front. Endocrinol..

[147] Yavuzer H., Yavuzer S., Cengiz M., Erman H., Doventas A., Balci H., Erdincler D.S., Uzun H. (2016). Biomarkers of lipid peroxidation related to hypertension in aging. Hypertens. Res..

[148] Duchnowicz P., Nowicka A., Koter-Michalak M., Broncel M. (2012). In vivo influence of extract from Aronia melanocarpa on the erythrocyte membranes in patients with hypercholesterolemia. Med. Sci. Monit..

[149] Pilaczynska-Szczesniak L., Skarpanska-Steinborn A., Deskur E., Basta P., Horoszkiewicz-Hassan M. (2005). The Influence of Chokeberry Juice Supplementation on the Reduction of Oxidative Stress Resulting from an Incremental Rowing Ergometer Exercise. Int. J. Sport Nutr. Exerc. Metab..

[150] Stojković L., Zec M., Zivkovic M., Bundalo M., Bošković M., Glibetić M., Stankovic A. (2021). Polyphenol-Rich Aronia melanocarpa Juice Consumption Affects LINE-1 DNA Methylation in Peripheral Blood Leukocytes in Dyslipidemic Women. Front. Nutr..

[151] Pearce M.S., McConnell J.C., Potter C., Barrett L.M., Parker L., Mathers J.C., Relton C.L. (2012). Global LINE-1 DNA methylation is associated with blood glycaemic and lipid profiles. Int. J. Epidemiol..

[152] Martín-Núñez G.M., Rubio-Martín E., Cabrera-Mulero R., Rojo-Martínez G., Olveira G., Valdés S., Soriguer F., Castaño L., Morcillo S. (2014). Type 2 diabetes mellitus in relation to global LINE-1 DNA methylation in peripheral blood: A cohort study. Epigenetics.

[153] Cash H.L., McGarvey S.T., Houseman E.A., Marsit C.J., Hawley N.L., Lambert-Messerlian G.M., Viali S., Tuitele J., Kelsey K.T. (2011). Cardiovascular disease risk factors and DNA methylation at the*LINE-1*repeat region in peripheral blood from Samoan Islanders. Epigenetics.

[154] Pan M.-H., Lai C.-S., Wu J.-C., Ho C.-T. (2013). Epigenetic and Disease Targets by Polyphenols. Curr. Pharm. Des..

[155] Liu G., Bin P., Wang T., Ren W., Zhong J., Liang J., Hu C.-A.A., Zeng Z., Yin Y. (2017). DNA Methylation and the Potential Role of Methyl-Containing Nutrients in Cardiovascular Diseases. Oxidative Med. Cell. Longev..

[156] Xu W., Larbi A. (2018). Immunity and Inflammation: From Jekyll to Hyde. Exp. Gerontol..

[157] Kotas M.E., Medzhitov R. (2015). Homeostasis, Inflammation, and Disease Susceptibility. Cell.

[158] Furman D., Campisi J., Verdin E., Carrera-Bastos P., Targ S., Franceschi C., Ferrucci L., Gilroy D.W., Fasano A., Miller G.W. (2019). Chronic inflammation in the etiology of disease across the life span. Nat. Med..

[159] Zhu Y., Xian X., Wang Z., Bi Y., Chen Q., Han X., Tang D., Chen R. (2018). Research Progress on the Relationship between Atherosclerosis and Inflammation. Biomolecules.

[160] Golia E., Limongelli G., Natale F., Fimiani F., Maddaloni V., Pariggiano I., Bianchi R., Crisci M., D’Acierno L., Giordano R. (2014). Inflammation and Cardiovascular Disease: From Pathogenesis to Therapeutic Target. Curr. Atheroscler. Rep..

[161] Lontchi-Yimagou E., Sobngwi E., Matsha T.E., Kengne A.P. (2013). Diabetes Mellitus and Inflammation. Curr. Diabetes Rep..

[162] Koyama Y., Brenner D.A. (2017). Liver inflammation and fibrosis. J. Clin. Investig..

[163] Kimura T., Isaka Y., Yoshimori T. (2017). Autophagy and kidney inflammation. Autophagy.

[164] Stephenson J., Nutma E., Van Der Valk P., Amor S. (2018). Inflammation in CNS neurodegenerative diseases. Immunology.

[165] Banerjee S., Biehl A., Gadina M., Hasni S., Schwartz D. (2017). JAK–STAT Signaling as a Target for Inflammatory and Autoimmune Diseases: Current and Future Prospects. Drugs.

[166] Chen L., Deng H., Cui H., Fang J., Zuo Z., Deng J., Li Y., Wang X., Zhao L. (2018). Inflammatory responses and inflammation-associated diseases in organs. Oncotarget.

[167] Arulselvan P., Fard M.T., Tan W.S., Gothai S., Fakurazi S., Norhaizan M.E., Kumar S.S. (2016). Role of Antioxidants and Natural Products in Inflammation. Oxid. Med. Cell. Longev..

[168] Menzel A., Samouda H., Dohet F., Loap S., Ellulu M.S., Bohn T. (2021). Common and Novel Markers for Measuring Inflammation and Oxidative Stress Ex Vivo in Research and Clinical Practice—Which to Use Regarding Disease Outcomes?. Antioxidants.

[169] Appel K., Meiser P., Millán E., Collado J.A., Rose T., Gras C.C., Carle R., Muñoz E. (2015). Chokeberry (*Aronia melanocarpa* (Michx.) Elliot) concentrate inhibits NF-κB and synergizes with selenium to inhibit the release of pro-inflammatory mediators in macrophages. Fitoterapia.

[170] Iwashima T., Kudome Y., Kishimoto Y., Saita E., Tanaka M., Taguchi C., Hirakawa S., Mitani N., Kondo K., Iida K. (2019). Aronia berry extract inhibits TNF-α-induced vascular endothelial inflammation through the regulation of STAT3. Food Nutr. Res..

[171] Ailuno G., Zuccari G., Baldassari S., Lai F., Caviglioli G. (2021). Anti-Vascular Cell Adhesion Molecule-1 Nanosystems: A Promising Strategy Against Inflammatory Based Diseases. J. Nanosci. Nanotechnol..

[172] Witkowska A.M., Borawska M.H. (2004). Soluble intercellular adhesion molecule-1 (sICAM-1): An overview. Eur. Cytokine Netw..

[173] Lin J., Kakkar V., Lu X. (2014). Impact of MCP -1 in Atherosclerosis. Curr. Pharm. Des..

[174] Wiesner P., Tafelmeier M., Chittka D., Choi S.-H., Zhang L., Byun Y.S., Almazan F., Yang X., Iqbal N., Chowdhury P. (2013). MCP-1 binds to oxidized LDL and is carried by lipoprotein(a) in human plasma. J. Lipid Res..

[175] Milne G.L., Musiek E.S., Morrow J.D. (2005). F_2_-Isoprostanes as markers of oxidative stress in vivo: An overview. Biomarkers.

[176] Proudfoot J.M., Murrey M.W., McLean S., Greenland E.L., Barden A.E., Croft K.D., Galano J.-M., Durand T.A., Mori T., Pixley F.J. (2018). F2-isoprostanes affect macrophage migration and CSF-1 signalling. Free Radic. Biol. Med..

[177] Milosavljevic I., Jakovljevic V., Petrovic D., Draginic N., Jeremic J., Mitrovic M., Zivkovic V., Srejovic I., Stojic V., Bolevich S. (2021). Standardized Aronia melanocarpa extract regulates redox status in patients receiving hemodialysis with anemia. Mol. Cell. Biochem..

[178] Serafini M., Peluso I. (2016). Functional Foods for Health: The Interrelated Antioxidant and Anti-Inflammatory Role of Fruits, Vegetables, Herbs, Spices and Cocoa in Humans. Curr. Pharm. Des..

[179] Huang J.-Q., Zhou J.-C., Wu Y.-Y., Ren F.-Z., Lei X.G. (2018). Role of glutathione peroxidase 1 in glucose and lipid metabolism-related diseases. Free Radic. Biol. Med..

[180] Lubos E., Loscalzo J., Handy D.E. (2011). Glutathione Peroxidase-1 in Health and Disease: From Molecular Mechanisms to Therapeutic Opportunities. Antioxid. Redox Signal..

[181] Wu J.H., Batist G. (2013). Glutathione and glutathione analogues; Therapeutic potentials. Biochim. Biophys. Acta.

[182] Loeffen R., Spronk H.M.H., Cate H.T. (2012). The impact of blood coagulability on atherosclerosis and cardiovascular disease. J. Thromb. Haemost..

[183] Seegers W.H. (1967). Blood Clotting Enzymology.

[184] Vita J.A. (2005). Polyphenols and cardiovascular disease: Effects on endothelial and platelet function. Am. J. Clin. Nutr..

[185] Bijak M., Bobrowski M., Borowiecka M., Podsędek A., Golański J., Nowak P. (2011). Anticoagulant effect of polyphenols-rich extracts from black chokeberry and grape seeds. Fitoterapia.

[186] Malinowska J., Babicz K., Olas B., Stochmal A., Oleszek W. (2012). Aronia melanocarpa extract suppresses the biotoxicity of homocysteine and its metabolite on the hemostatic activity of fibrinogen and plasma. Nutrition.

[187] Sikora J., Markowicz-Piasecka M., Broncel M., Mikiciuk-Olasik E. (2014). Extract of *Aronia melanocarpa*-modified hemostasis: In vitro studies. Eur. J. Nutr..

[188] Stevanović V., Pantović A., Krga I., Zeković M., Šarac I., Glibetić M., Vidović N. (2020). Aronia juice consumption prior to half-marathon race can acutely affect platelet activation in recreational runners. Appl. Physiol. Nutr. Metab..

[189] Arias I.M., Alter H.J., Boyer J.L., Cohen D.E., Shafritz D.A., Thorgeirsson S.S., Wolkoff A.W. (2020). The Liver: Biology and Pathobiology.

[190] Mansouri A., Gattolliat C.-H., Asselah T. (2018). Mitochondrial Dysfunction and Signaling in Chronic Liver Diseases. Gastroenterology.

[191] Ceriotti F., Henny J., Queraltó J., Ziyu S., Özarda Y., Chen B., Boyd J.C., Panteghini M. (2010). Common reference intervals for aspartate aminotransferase (AST), alanine aminotransferase (ALT) and γ-glutamyl transferase (GGT) in serum: Results from an IFCC multicenter study. Clin. Chem. Lab. Med..

[192] Li S., Tan H.-Y., Wang N., Zhang Z.-J., Lao L., Wong C.-W., Feng Y. (2015). The Role of Oxidative Stress and Antioxidants in Liver Diseases. Int. J. Mol. Sci..

[193] Li S., Tan H.Y., Wang N., Cheung F., Hong M., Feng Y. (2018). The Potential and Action Mechanism of Polyphenols in the Treatment of Liver Diseases. Oxid. Med. Cell. Longev..

[194] Mężyńska M., Brzóska M.M., Rogalska J., Piłat-Marcinkiewicz B. (2018). Extract from Aronia melanocarpa L. Berries Prevents Cadmium-Induced Oxidative Stress in the Liver: A Study in A Rat Model of Low-Level and Moderate Lifetime Human Exposure to this Toxic Metal. Nutrients.

[195] Park C.-H., Kim J.-H., Lee E.B., Hur W., Kwon O.-J., Park H.-J., Yoon S.K. (2017). Aronia melanocarpa Extract Ameliorates Hepatic Lipid Metabolism through PPARγ2 Downregulation. PLoS ONE.

[196] Yang J., Gao J., Yu W., Hao R., Fan J., Wei J. (2020). The effects and mechanism of Aronia melanocarpa Elliot anthocyanins on hepatic fibrosis. J. Funct. Foods.

[197] Kozłowska M., Brzóska M.M., Rogalska J., Galicka A. (2020). The Impact of a Polyphenol-Rich Extract from the Berries of *Aronia melanocarpa* L. on Collagen Metabolism in the Liver: A Study in an In Vivo Model of Human Environmental Exposure to Cadmium. Nutrients.

[198] Glantzounis G.K., Tsimoyiannis E.C., Kappas A.M., Galaris D.A. (2005). Uric Acid and Oxidative Stress. Curr. Pharm. Des..

[199] Wu A.H., Gladden J.D., Ahmed M., Ahmed A., Filippatos G. (2016). Relation of serum uric acid to cardiovascular disease. Int. J. Cardiol..

[200] Srivastava A., Kaze A.D., McMullan C.J., Isakova T., Waikar S.S. (2018). Uric Acid and the Risks of Kidney Failure and Death in Individuals With CKD. Am. J. Kidney Dis..

[201] Yanai H., Adachi H., Hakoshima M., Katsuyama H. (2021). Molecular Biological and Clinical Understanding of the Pathophysiology and Treatments of Hyperuricemia and Its Association with Metabolic Syndrome, Cardiovascular Diseases and Chronic Kidney Disease. Int. J. Mol. Sci..

[202] Wang J., Qin T., Chen J., Li Y., Wang L., Huang H., Li J. (2014). Hyperuricemia and Risk of Incident Hypertension: A Systematic Review and Meta-Analysis of Observational Studies. PLoS ONE.

[203] Lv Q., Meng X.-F., He F.-F., Chen S., Su H., Xiong J., Gao P., Tian X.-J., Liu J.-S., Zhu Z.-H. (2013). High Serum Uric Acid and Increased Risk of Type 2 Diabetes: A Systemic Review and Meta-Analysis of Prospective Cohort Studies. PLoS ONE.

[204] Chen S., Yang H., Chen Y., Wang J., Xu L., Miao M., Xu C. (2020). Association between serum uric acid levels and dyslipidemia in Chinese adults: A cross-sectional study and further meta-analysis. Medicine.

[205] Sautin Y.Y., Johnson R.J. (2008). Uric Acid: The Oxidant-Antioxidant Paradox. Nucleosides Nucleotides Nucleic Acids.

[206] Lippi G., Montagnana M., Franchini M., Favaloro E.J., Targher G. (2008). The paradoxical relationship between serum uric acid and cardiovascular disease. Clin. Chim. Acta.

[207] Li L., Li J., Xu H., Zhu F., Li Z., Lu H., Zhang J., Yang Z., Liu Y. (2021). The Protective Effect of Anthocyanins Extracted from Aronia Melanocarpa Berry in Renal Ischemia-Reperfusion Injury in Mice. Mediat. Inflamm..

[208] Delanaye P., Cavalier E., Pottel H. (2017). Serum Creatinine: Not So Simple!. Nephron.

[209] Khosla N., Sarafidis P.A., Bakris G.L. (2006). Microalbuminuria. Clin. Lab. Med..

[210] Savarese G., Dei Cas A., Rosano G., D’Amore C., Musella F., Mosca S., Reiner M.F., Marchioli R., Trimarco B., Perrone-Filardi P. (2014). Reduction of albumin urinary excretion is associated with reduced cardiovascular events in hypertensive and/or diabetic patients. A meta-regression analysis of 32 randomized trials. Int. J. Cardiol..

[211] Hong Z., Jiang Y., Liu P., Zhang L. (2021). Association of microalbuminuria and adverse outcomes in hypertensive patients: A meta-analysis. Int. Urol. Nephrol..

